# Cultural Competency in Research: A Practical Framework for Use by Researchers, Policymakers, Community Leads and Others When Working With People From Diverse Groups

**DOI:** 10.1111/hex.70544

**Published:** 2026-01-13

**Authors:** Evgenia Stepanova, Matthew Cooper, Anna Robinson‐Barella, Vicki Harris, Abbie Rance, Abbie Husband, David Wright, Fabi Lorencatto, Clare Tolley, Wade Tovey, Geoff Wong, Daniel Cowie, Kerry Parker, Elise Crayton, Hamde Nazar

**Affiliations:** ^1^ National Institute for Health and Care Research Newcastle Patient Safety Research Collaboration Newcastle University Newcastle upon Tyne UK; ^2^ School of Pharmacy Newcastle University Newcastle upon Tyne UK; ^3^ Newcastle Health Research Partnership & Clinical Research Directorate Newcastle upon Tyne Hospitals NHS Foundation Trust Newcastle upon Tyne UK; ^4^ Connected Voice Newcastle upon Tyne UK; ^5^ School of Healthcare University of Leicester Leicester UK; ^6^ Department of Clinical, Educational and Health Psychology, Centre for Behaviour Change University College London London UK; ^7^ North East Social Care Advisors CIC Newcastle upon Tyne UK; ^8^ Nuffield Department of Primary Care Health Sciences University of Oxford Oxford UK; ^9^ NHS North East and North Cumbria Integrated Care Board Newcastle upon Tyne UK; ^10^ Age UK North Tyneside North Shields UK

**Keywords:** cultural competence, cultural humility, inclusive research, minorities, racialised communities

## Abstract

**Background:**

As researchers strive to conduct culturally competent and equitable research, there remains a lack of actionable guidance across the research lifecycle. Increasingly, funding bodies require evidence of such approaches, yet a clear, practical direction for effective research practice is often lacking.

**Aim:**

To develop a practical framework, grounded in Meleis' cultural competence criteria, to guide all stages of the research lifecycle.

**Methods:**

Databases PsycINFO, ERIC, PubMed, Web of Science Core Collection and Google Scholar were searched (August–October 2025) for English‐language publications applying Meleis' framework in research contexts. Narrative and thematic analysis synthesised identified applications into draft recommendations and measures across five research stages. A modified Delphi process with 31 experts (community leads, researchers, policymakers and translators) in two survey rounds and a consensus workshop was then used to refine and finalise items, producing a co‐developed framework of actionable, measurable recommendations for culturally competent research with minoritised and/or diverse groups.

**Results:**

The review identified eight studies, yielding 41 applications of Meleis' criteria. These were distilled into 29 draft recommendations and measures, which were further refined through Delphi consensus to 25 recommendations and 25 measures, offering practice‐ready guidance across research focus, recruitment, measurement, analysis and dissemination. Contextuality was rated critical in four stages, highlighting how cultural identities shape engagement. Language was strongly prioritised in focus, recruitment and measurement, with emphasis on diverse, well‐funded interpretation and transcription strategies. Empowerment and reciprocation were noted as underused but essential for legitimacy and impact, particularly in focus and dissemination. Continuous cultural humility training was endorsed, reinforcing critiques of competence as a static skill. During consensus discussions, recommendations initially framed as optional (‘could’) were reframed as compulsory (‘should’). The final framework is available as an accessible online resource.

**Conclusions:**

Using consensus, an evidence‐based, culturally competent research framework was co‐designed; it comprised 25 recommendations that are actionable, measurable and adaptable by researchers, policymakers, community leads and others. This framework represents a crucial step towards fostering equitable and impactful research practice.

**Patient or Public Contribution:**

A patient and public involvement group of three patients from minoritised backgrounds and two health professionals met twice to review findings, refine components and provide feedback on language, accessibility and usability.

## 1 Introduction

Cultural competency has been explored across healthcare [1, 2], education [3], social work [4] and psychology [5], leading to frameworks that emphasise knowledge, attitudes, skills and, increasingly, systemic and organisational change. It is often defined as ‘*a set of congruent behaviours, knowledge, attitudes … that enables effective work in cross‐cultural situations*’ [6].

Across the research lifecycle, however, there has been continuous oversight on cultural competency and equity of practice [7]. This has resulted in ongoing under‐representation of diverse populations and contributed to inequities for those from marginalised and/or minoritised groups [8, 9]. Cultural competency studies have previously excluded non‐English speakers, relied on materials that are linguistically or culturally inaccessible, failed to engage with trusted community partners, and under‐represented populations; this has led to biases that differentially distribute opportunities and harms. These findings contribute to a broader problem of epistemic injustice where some groups lack equal roles in the production, interpretation and application of research. Addressing this injustice requires research practices that redistribute epistemic structure and ensure that diverse populations can meaningfully shape and inform research strategy, processes, decisions and outcomes [7].

Policy frameworks, such as the NHS Migrant Health Guide [10], the U.S. CLAS Standards [11] and Australia's Cultural Competency in Health [12], have aimed to embed cultural competence in health systems. However, their application in research is limited. Guidelines have previously been underused or poorly integrated, or lack enforcement among research teams [13]. They have rarely been integrated into ethics review, protocol development or evaluation metrics, and where referenced, inclusion efforts have frequently been overstated; for example, including diversity within participant groups but without adapting the research methodology to their individual, cultural or linguistic needs [9, 14].

Practically, health and social care professionals, researchers, and service leads may have previously lacked the knowledge, communication strategies and cultural awareness needed to work effectively in cross‐cultural contexts [15, 16]. Such gaps have contributed to persistent inequities in how research is conducted and how services are designed and delivered. Without a strong grounding in cultural competence, research may have unintentionally generalised, stereotyped or misrepresented people from culturally and linguistically diverse populations, compromising both ethical integrity and research validity [17, 18].

While clinical and educational settings have increasingly adopted culturally competent frameworks [19], such approaches are far less embedded within health and social research practice. In general, they were developed to strengthen practitioner–patient interactions and professional training. For example, in clinical practice, the existing frameworks inform care planning and strengthen communication across cultural contexts [3, 20]. In educational settings, they underpin curriculum development, teaching of transcultural skills and evaluation of student cultural competence [20, 21].

Researchers have described receiving limited training or support on how to meaningfully incorporate equality, diversity and inclusion (EDI) into all stages of the research process—in turn, this has resulted in a disconnect between theoretical awareness of cultural competence and its practical application. For example, recruitment strategies may have failed to reach diverse populations due to a lack of engagement with trusted community organisations or the use of exclusionary eligibility criteria (e.g., requiring fluency in the English language or having access to digital technologies) [22]. In designing and choosing methodology, researchers may have overlooked the need for culturally tailored interview techniques, multilingual resources or appropriate interpreters [2]. During analysis, findings may have been interpreted through a dominant cultural lens, without adequate contextualisation of cultural beliefs, norms or values [23]. Similarly, research dissemination has often failed to include accessible formats or community feedback loops, which has led to further marginalisation of those already under‐represented [8].

Too often, cultural considerations have been treated as afterthoughts—addressed in post hoc reflections or tokenistic involvement of community representatives—rather than embedded as a core methodological principle throughout the research lifecycle [9, 24]. This has not only undermined inclusion but also reinforced systemic barriers for marginalised groups to participate in or benefit from research and service improvement efforts.

While there has been growing recognition of the need for equity in research involving minoritised communities [24, 25], there is currently no widely adopted framework to help researchers operationalise this. Without practical guidance, researchers risk reinforcing structural inequalities and excluding under‐represented groups [9, 24]. The NIHR INCLUDE Ethnicity Framework [25], Equator Network's reporting guidelines [26] and Community‐Based Participatory Research (CBPR) principles [27] have shown promise in improving inclusivity and relevance; however, their use remains inconsistent and not yet embedded across mainstream research practice.

## 2 PPIE Versus Cultural Competence Strategy

One way to help ensure that the principles of inclusion, contextuality and relevance are embedded in research is through Patient and Public Involvement and Engagement (PPIE). Many funders now require a dedicated PPIE section as an essential component of the research process, with PPIE defined as research carried out ‘with’ or ‘by’ patients and the public, rather than ‘to’, ‘about’ or ‘for’ them [28]. PPIE focuses on actively involving patients and public contributors in shaping research priorities, study design, analysis and dissemination to improve the relevance, accessibility and impact of research [29, 30].

In contrast, a cultural competence strategy emphasises the skills, knowledge and behaviours required to design and conduct research that is sensitive to cultural, linguistic and historical contexts, including the recognition of power dynamics and adaptation of communication styles to promote equitable participation [1, 16].

While both approaches aim to enhance inclusivity and the meaningfulness of research, PPIE centres on who is involved—ensuring that patients and the public shape the research agenda—whereas cultural competence focuses on how research is conducted to ensure cultural sensitivity and equity. Together, they represent complementary but distinct pathways for achieving inclusive, contextually responsive and impactful research.

## 3 From Theory to Practice: A Framework for Culturally Competent Research

Meleis' cultural competence framework, developed in the United States, was selected for its comprehensive, process‐oriented approach, emphasising critical self‐awareness, attention to power dynamics and meaningful community engagement across all phases of the research lifecycle [31]. Other internationally recognised models, such as Campinha‐Bacote's Process of Cultural Competence [20], Leininger's Cultural Care Theory [32], the Giger–Davidhizar Transcultural Assessment Model [33] and the U.S. Department of Health and Human Services' CLAS Standards [9] have primarily addressed clinical practice, communication and language accessibility, offering limited applicability to research design and conduct. The Purnell Model for Cultural Competence [34] and the Papadopoulos–Tilki–Taylor model [21] are also influential but are primarily operationalised in clinical education and service delivery rather than research design. In a UK context, the adapted Gibbs et al. framework sets out nine criteria for engagement, recruitment, analysis and dissemination in partnership with target populations but provides limited strategies for applying these principles in diverse research contexts [35]. The EqIA Toolkit supports compliance with the UK Equality Act 2010, yet does not address the deeper cultural, linguistic or historical contexts essential for cultural competence [36].

The NIHR Race Equality Framework offers organisational self‐assessment across five domains—individual responsibility, leadership, public partnerships, recruitment and systems/processes—but lacks explicit guidance for iterative adaptation during research [37]. The multicultural research framework by Woodland et al. strengthens ethical practice, trust‐building and communication, yet omits sustained engagement with researcher positionality or structural inequities [38]. Additionally, the NIHR INCLUDE Ethnicity Framework [25] and the EQUATOR Network's reporting guidelines [26] have shown promise in improving inclusivity and relevance; however, their use remains inconsistent and is not yet embedded across mainstream research practice.

In contrast, Meleis' framework [31] uniquely integrates individual, relational and contextual dimensions, making it particularly effective for embedding culturally responsive, ethically rigorous research practices across all stages of the research [1]. However, Meleis' framework—while conceptually clear—was originally designed for nursing and educational contexts, not research. Although some scholars have broadened its application [39], the framework still lacks concrete procedural guidance to address the methodological complexities inherent in the research process; for example, ensuring methodological rigour in diverse settings, navigating ethical tensions in co‐production or balancing institutional requirements with community priorities [40]. This limitation is reflected in the framework's limited uptake outside health and education disciplines [41].

To address this gap, this work aims to operationalise Meleis' criteria into a structured and accessible framework, co‐designed in partnership with researchers, community leads, translators and interpreters, and policymakers from diverse backgrounds. This culturally competent research framework is intended to move beyond theoretical intention, supporting the practical application of culturally competent research across disciplines and sectors, as well as the research lifecycle.

**Figure 1:**
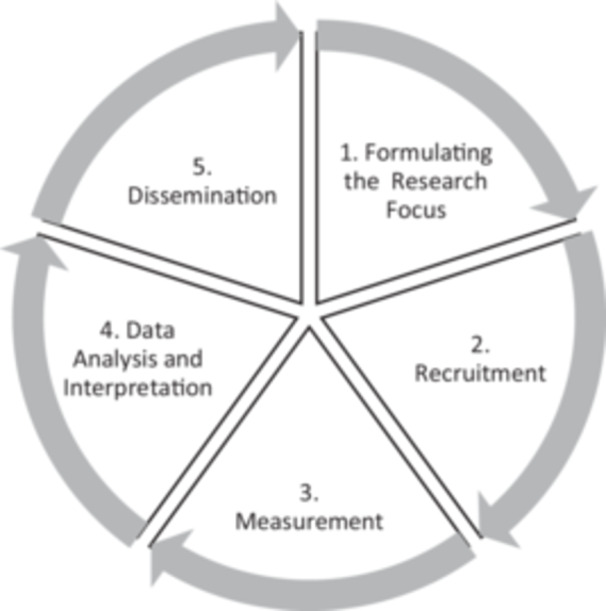
The research lifecycle.

The research lifecycle in this study includes five methodological stages: research problem formulation, recruitment, measurement, interpretation and dissemination [42, 43] (Figure 1).

## 4 Materials and Methods

### 4.1 Study Design

The study design included three phases: a rapid review, a thematic analysis of rapid review data and a modified Delphi study, which included two rounds of a survey and a consensus workshop to finalise the framework.

#### 4.1.1 Phase 1

To inform the initial list of suggested recommendations and measures, a rapid review of the literature on Meleis' culturally competent framework was conducted. The review followed principles outlined in the Cochrane Rapid Reviews Interim Guidance [44], enabling synthesis of relevant literature while maintaining transparency and methodological rigour.

#### 4.1.2 Phase 2

Following the review, the identified applications were examined and refined through study team meetings using thematic analysis. Each application of Meleis' criteria was systematically assessed and translated into draft recommendations aligned with core aspects of cultural competence. Overlapping narratives were synthesised into broader recommendations. For each recommendation, a corresponding measure was then developed to translate the recommendation into an assessable action and to support evaluation of its implementation. Measures were explicitly derived from the content and intent of each recommendation and were framed as practical, reflective questions to determine whether the recommended behaviour had been enacted in practice. For example, the recommendation ‘*Researchers need to consider building direct relationships with the community in focus’* was operationalised through the measure ‘*Have the researchers considered ways of building direct relationships with the community in focus through activities beyond those directly related to the research?’*

#### 4.1.3 Phase 3

We employed a modified Delphi study to build consensus on recommendations and measures, grounded in Meleis' cultural competence criteria, for application across the research lifecycle. Here, recommendations refer to specific action points designed to translate culturally competent principles into practice, while measures denote the means of verifying that these behaviours have been implemented. The Delphi method, a structured and iterative process for collecting and refining expert opinion, is particularly well suited for areas where evidence is emerging and prescriptive guidance is limited [45]. Two key modifications were made to the conventional Delphi method: first, rather than beginning with open‐ended expert input, we initiated the process with the framework derived from a preceding rapid literature review. This evidence‐informed starting point is seen as a more advanced and streamlined process, and focused efforts on refining and prioritising actionable, meaningful content [46]. Second, the process concluded with a consensus workshop designed to enable dialogue and address any remaining areas of divergence.

#### 4.1.4 Survey Round 1

In Round 1, the survey—hosted on the OnlineSurveys platform [47]—included a participant information sheet, consent form, demographic questions and the full set of statements and measures, organised by the five stages of the research process: (1) formulating the research focus, (2) recruitment, (3) measurement, (4) data analysis and interpretation and (5) dissemination. Participants rated each item on a 5‐point Likert scale (1 = ‘Not at all important’ to 5 = ‘Extremely important’) and could suggest additional items via optional free‐text fields. Invitations were distributed via email, with up to three reminders issued to optimise response rates.

Round 1 responses were analysed using both quantitative and qualitative methods. Items—both recommendations and measures—were considered to have achieved consensus if at least 85% of participants rated them as ‘Very important’ or ‘Extremely important’ (scores of 4 or 5), with median scores also calculated for each item. This threshold aligns with reported Delphi consensus ranges of 50%–97% [48] and is consistent with previous research that uses higher thresholds (≥ 85%) to increase the robustness of agreement, particularly when validating scale items or defining best practice [49].

A median score of 5 was used as a complementary indicator of strong agreement and to validate consensus findings. Free‐text feedback was analysed using content analysis and informed the revision or supplementation of the item set for Round 2.

#### 4.1.5 Survey Round 2

The Round 2 survey presented the revised set of recommendations and measures, alongside summarised Round 1 results, including the median score and three comparison charts per item (full panel, community leaders/policymakers and researchers). This structured feedback allowed participants to consider the broader group's views and revise their ratings accordingly. To minimise respondent burden, free‐text fields were omitted in this round. The survey also included reconfirmation of consent and demographic information. All Round 1 participants were invited to complete Round 2.

### 4.2 Consensus Workshop

Following the completion of both survey rounds, participants were invited to a virtual consensus workshop, conducted via Microsoft Teams, to enable broad geographic participation. The workshop was recorded and transcribed. Participants received a pre‐circulated workshop booklet summarising the Delphi survey findings to facilitate discussion. The workshop aimed to address partial agreement, clarify ambiguities and finalise the structure and content of the framework.

### 4.3 Defining Consensus

Consensus in Delphi studies is not defined by a universal standard; thresholds in the literature range from 50% to 97% [50], depending on the rating scale and methodological approach. For this study, a consensus threshold of 85% agreement on a five‐point Likert scale was adopted, aligning with thresholds used in previous research [49]. In addition to percentage agreement, median scores were calculated to capture the central tendency of responses, as medians are less affected by outliers or skewed distributions than means. A median score of 5 was used as a complementary indicator of strong agreement and to validate consensus findings.

Ethical approval was granted by the Ethics Committee at the School of Pharmacy, Newcastle University (Ref: 51189/2023).

### 4.4 Participants and Recruitment

Modified Delphi studies aimed at developing consensus should strive to include ‘experts’ who are appropriate to the subject matter and are credible representatives of the target audience [49]. Particular attention was paid to recruiting individuals with direct experience of working with minoritised, linguistically diverse and structurally marginalised populations. The panel was intentionally composed of four key stakeholder groups: community leaders, policymakers, researchers and translators across multidisciplinary domains, including health, social work and others (see Table 2 for further details). Community leaders were identified through local organisations partnered with the project team. Policymakers and researchers were recruited through established networks within Cumbria, Northumberland, Tyne and Wear NHS Foundation Trust hospitals. Translators were recruited via professional translation agencies [51]. Additionally, recruitment was supplemented through targeted outreach on social media platforms (Facebook). Inclusion criteria prioritised panellists' experiential and professional expertise in culturally responsive research practice so that individual demographic characteristics such as ethnicity, age or gender were not used as selection criteria. Patients and carers were not invited to participate in the Delphi panel, as the primary aim of the study was to develop a set of transferable, methodological recommendations applicable across diverse research contexts rather than to focus on condition or population‐specific lived experiences. Instead, the Delphi process was designed to draw on expert perspectives with cross‐sectoral and cross‐population insight into research design, governance, ethics and implementation. Adults able to use/access the Internet and email were eligible for inclusion if they had relevant experience listed in Table 2.

**Table 1:** Summary of the key criteria of culturally competent research based on Meleis' (1996) Culturally Competent Scholarship [31].

Criterion	Explanation
Contextuality	Knowledge must be situated in the socio‐cultural, historical and political context of those involved; meanings are derived from that lived context rather than abstracted from it.
Relevance	The focus and outcomes should be significant to the people and communities concerned, reflecting their priorities and concerns.
Communication styles	Scholarly work should be congruent with participants' linguistic and non‐linguistic communication patterns, respecting culturally shaped ways of expressing meaning.
Awareness of identity and power differentials	Scholars must recognise their own and others' identities and the power hierarchies present, explicitly addressing how these shape interactions and knowledge production.
Disclosure	Openness and clarity about aims, roles, methods, risks/benefits and positions are essential to maintain integrity and trust.
Reciprocation	There should be mutual exchange and benefit; scholarship should avoid one‐sided extraction and acknowledge contributions in culturally appropriate ways.
Empowerment	Work should enhance people's sense of agency and options, supporting greater control over decisions that affect their lives.
Time	Adequate and flexible time is required to build relationships and achieve meaningful outcomes, with sensitivity to cultural orientations to time and pacing.

**Table 2:** Inclusion criteria of expert stakeholders.

Stakeholder group	Inclusion criteria
Community leads	Interest and experience in working with ethnic minorities and linguistically diverse populations.
Policymakers	Professional experience in planning and implementing services for ethnic minorities and linguistically diverse populations.
Researchers	Experience in conducting and managing research with and around ethnic minorities and linguistically diverse populations.
Translators	Professional experience in providing translation/interpretation services for research purposes.

There are no universally agreed‐upon guidelines regarding the optimal size of Delphi panels [49, 52]. For this study, a target range of 15–30 panellists was established to balance inclusivity with feasibility. This size range allows for diverse stakeholder representation and balanced distribution—essential in culturally competent research—while also ensuring sufficient interaction, manageable data synthesis and participant retention across multiple Delphi rounds. A combined purposive and snowball sampling strategy was employed to enhance heterogeneity in perspectives and experiences [40, 53].

To encourage complete participation across all phases of the research, gift vouchers were provided upon completion of the second survey round.

### 4.5 Anonymity

While anonymity is often limited in Delphi studies due to their iterative design, participants in this study were given the option to remain anonymous, given the sensitivity of the topic.

### 4.6 Public Involvement and Engagement

To ensure the final framework reflected the lived experiences of, and was relevant to, minoritised populations, we embedded Public Involvement and Engagement (PIE) throughout the project. Two PIE groups included three public representatives from ethnically minoritised backgrounds and two health professionals working with ethnically minoritised communities. The meetings were facilitated by four members of the project team (E.S., A.R.‐B., M.C., A.H. and A.B.). Each group met twice to review emerging findings, refine framework components and provide feedback on language, accessibility and usability.

### 4.7 Analysis

#### 4.7.1 Phases 1 and 2

Guided by transparent and reproducible evidence synthesis, we selected both narrative and thematic analyses of the extracted data. The narrative analysis summarised key findings across studies, while thematic analysis, based on King et al. (2022) [54], categorised implementation strategies, challenges and adaptations of Meleis' criteria. Reflexive meetings among the research team were held to review the emerging themes and discuss how the recommendations and measures could be refined or expanded to support practical application.

#### 4.7.2 Phase 3

Survey data from both rounds were analysed quantitatively and qualitatively. Quantitative analysis included descriptive statistics for each item and participant demographics, using Microsoft Excel. Responses were examined collectively (all panellists) and by subgroup (community leaders/policymakers and researchers) to assess differential patterns of agreement across stakeholder types. Items that achieved consensus in either or both rounds were retained for inclusion in the final list of recommendations presented at the consensus workshop.

Qualitative analysis of Round 1 free‐text responses was conducted using directed content analysis [48], with the five stages of the research process serving as top‐level categories. These responses were incorporated into an initial codebook, developed collaboratively by three researchers, which documented the definitions, inclusion criteria and examples for each code. Individual items were coded as subcategories under the five stages, with additional subcategories generated inductively when responses did not align with the existing structure. These emergent codes informed the revision of existing statements and the development of new items for Round 2. Workshop data were analysed thematically using Braun and Clarke's approach [55], enabling identification of overarching themes and sub‐themes related to culturally competent strategies in research. Two researchers independently reviewed the transcripts and iteratively refined the workshop codebook, ensuring clear definitions and consistent application of codes. The resulting themes and sub‐themes provided further clarification of areas of partial agreement and guided final refinements to the framework.

## 5 Results

### 5.1 Phase 1

Eight studies met the inclusion criteria, spanning the disciplines of nursing, social work and health research. The studies focused on diverse populations, including Korean women [56, 57], language minorities [43, 58] and marginalised communities [59, 60, 61].

The publications were from the late 1990s to early 2000s, reflecting an early engagement with cultural competence in health‐related research. All included records were conducted in or affiliated with institutions in the United States, with some focusing on immigrant or minority communities originating from East Asia and Latin America.

Based on the included studies, 82 initial applications of Meleis' criteria were extracted. While the included studies referenced or applied Meleis' criteria, the presentation of cultural competence was typically narrative in form, lacking actionable recommendations or clearly defined measures.

### 5.2 Phase 2

Following the review, these 82 narratives were assessed and refined through a series of study team meetings guided by narrative and thematic analysis [55] to develop recommendations and measures. This process involved systematically examining each study's application of Meleis' eight criteria and transforming each application into recommendations that aligned with core aspects of cultural competence. During this stage, narratives were collapsed and merged together to form broad and encompassing recommendations. For each recommendation, a corresponding measure was created to operationalise the intended behaviour, providing a concrete means of assessing whether the recommended culturally competent practice had been implemented. This ensured that the measures not only reflected the recommendations but also served as practical tools for monitoring and evaluating their application in research. Drawing on the study findings, we extended Meleis' framework by adding an additional criterion of Language, resulting in a final set of nine criteria supported by 29 recommendations and 29 measures. Language emerged as a distinct and cross‐cutting theme that could not be fully captured within the existing criteria, ensuring its recognition and significant role as a standalone criterion of culturally competent research practice.

A detailed description of the rapid review process and study team discussion stages can be found in Supplementary File 1.

### 5.3 Phase 3

#### 5.3.1 Participants

Delphi participants (*n* = 31) represented a diverse mix of community leads (*n* = 12), researchers (*n* = 14), policymakers (*n* = 2) and translators/interpreters (*n* = 3), with 26 participants in Round 1, 24 in Round 2 and 14 in the final consensus workshop. Participants came from a range of ethnic backgrounds, including Asian or Asian British (*n* = 6), Black or Black British (*n* = 4), White (*n* = 12), Mixed (*n* = 2) and Other ethnic groups (*n* = 7).

Fourteen participants took part in a 2‐h online consensus workshop, including ten who continued from the survey—six researchers, four community leads and one policymaker—along with three newly joined translators. Thirty panellists had significant experience working with ethnically minoritised and linguistically diverse populations, with five reporting over 5 years of relevant experience and eight over 10 years. Table 3 provides the characteristics of the expert panel.

**Table 3:** Characteristics of the expert panel.

Panellists	Round 1: Survey (*n* = 26)	Round 2: Survey (*n* = 24)	Round 3: Consensus workshop (*n* = 14)
Type of expertise			
Community lead	11	12	4
Researcher	14	10	6
Policymaker	1	2	1
Translator/Interpreter	n/a	n/a	3
Ethnicity			
Asian or Asian British	6	6	3
Black, Black British, Caribbean or African	4	4	4
Mixed or multiple ethnic groups	1	1	0
Other ethnic groups	2	2	3
White or white British	12	11	4
Prefer not to say	1	0	0
Years of experience in supporting ethnic minority groups and linguistically diverse populations			
1–2	2	5	0
2–3	5	2	0
3–4	5	5	4
4–5	3	5	3
5–10	3	3	5
Less than 1 year	1	0	0
More than 10 years	7	4	2

In response to themes emerging from the surveys, additional participants with professional experience in interpretation and translation across various languages, specifically for research purposes, were invited to join the workshop.

## 6 Consensus Development

### 6.1 Survey Rounds 1 and 2

**Figure 2:**
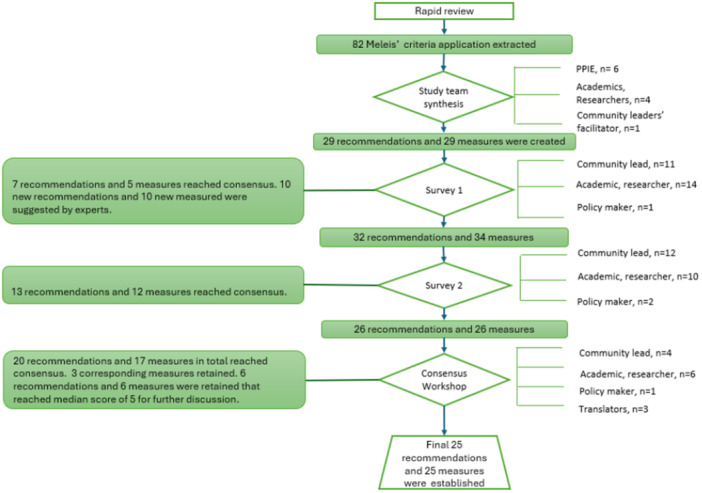
Modified Delphi process and results.

Based on a rapid review and study team synthesis, 29 recommendations and 29 measures were initially proposed (see Figure 2). The complete survey results are summarised in Table 4. Following Survey Round 1, seven recommendations and five measures achieved the consensus threshold. Furthermore, participant feedback via free‐text responses generated 10 new recommendations and 10 new measures, resulting in 32 recommendations and 34 measures for Round 2. Critically, contextuality and language criteria were prioritised throughout Round 1, highlighting the importance of participants' experiences of culture concerning how they feel, think, express, behave and how this may influence data being collected.

**Table 4:** Final list of recommendations from Survey Rounds 1 and 2.

			Community leads, (%)			Researchers, (%)			Policymakers, (%)			All, (%)			Consensus reached	
Research stage	Criteria	Recommendation	1–2	3	4–5	1–2	3	4–5	1–2	3	4–5	1–2	3	4–5	S1	S2
Research Stage 1: Formulating the area of research focus and interest	Contextuality	Researchers could seek to study populations' experiences of culture about how they feel, think, express and behave within the phenomenon/area of interest and how this may underpin their perspectives, expressions and behaviours.	0	16.7	75	0	10	90	0	0	100	0	12.5	87.5		√
	Contextuality	Researchers could conduct a literature review initially to establish which voices of the study population have already been explored.	0	33.3	66.7	10	50	40	0	50	50	4.2	41.6	54.1		
	Relevance	Researchers could engage with community leaders who can provide culturally sensitive information to support research problem formulation.	8.3	0	91.7	10	10	80	0	0	100	8.3	4.2	87.5		√
	Relevance	Researchers could include a patient/public member in the research team to guide culturally sensitive problem formulation.	0	8.3	91.7	0	0	100	0	0	100	0	4.2	95.8		√
	Reciprocation	Researchers could ask communities involved in the research what forms of enrichment beyond monetary compensation participants would prefer.	0	25	75	0	30	70	0	50	50	0	29.1	70.8		
	Empowerment	Researchers could show evidence to demonstrate their appreciation of power dynamics between them and different groups and communities involved.	8.3	41.7	50	10	20	70	0	0	100	8.3	29.1	62.5		
	Language	Researchers could enquire about the need for interpretation and/or translation services.	0	0	100	0	7.2	92.8	0	0	100	0	11.5	88.5	√	
	Contextuality	The researchers could undergo cultural competency training.	8.3	8.3	83.4	0	20	80	0	0	100	4.2	12.5	83.3		
	Contextuality	Researchers could align the area of research focus with research sensitivity that respects the study populations' age, sexual orientation, cultural heritage, social class, work situation and gender inequity.	9.1	9.1	81.8	7.2	0	92.8	0	0	100	7.7	3.8	88.5	√	
	Relevance, Awareness of Identity and Power Differentials	New: Researchers could consider the development of a patient/public involvement strategy to ensure their impact is useful, productive, appropriate and balanced in relation to the ratio (PPIE/researchers).	0	25	75	0	50	50	0	0	100	0	33.3	66.7		
	Relevance	New: Researchers should have a strategy to capture and monitor the impact and value of patient and public involvement.	0	8.3	91.7	0	10	90	0	0	100	0	8.3	91.7		√
	Contextuality	New: Researchers need to consider building direct relationships with the community in focus.	0	0	100	0	20	80	0	0	100	0	8.3	91.7		√
	Language	New: Researchers could consider the need for additional funding dedicated to covering costs for translation and interpretation.	0	8.3	91.7	0	20	80	0	0	100	0	8.3	91.7		√
Research Stage 2: Recruitment	Communication Style	Researchers could develop a communication strategy tailored to community involvement.	0	11.2	88.8	7.2	0	92.8	0	0	100	3.8	7.7	88.5	√	
	Time	Researchers could demonstrate flexibility in their strategy to reimburse and/or incentivise participants for their time.	0	0	100	0	30	70	0	50	50	0	16.6	83.4		
	Contextuality	Researchers could engage with a trusted/respected person within their field/community who can provide culturally sensitive information to support study recruitment.	0	8.3	91.7	0	0	100	0	0	100	0	0	100		√
	Language	Researchers could consider the use of interpretation and/or translation services.	0	9.1	90.9	0	7.2	92.8	0	0	100	3.8	7.7	88.5	√	
	Relevance	Researchers could demonstrate how they have tailored recruitment and facilitated a diverse representation of the community.	0	16.6	83.4	0	10	90	0	0	100	0	25	75		
	Empowerment	Researchers could consider strategies for empowering participants that go beyond financial incentivisation (e.g., Researchers to provide an ability to demonstrate connectedness to the research and a sense of freedom in modifying any parts of the process).	16.6	0	83.4	0	20	80	0	0	100	8.3	8.3	83.4		
	Disclosure	Ethics should be discussed through the lens of ethnicity and culture, including provision of culturally appropriate study explanations, assessing the cultural impediments to what researchers consider truly informed consent or the degree to which it is achieved, considering the ratio of risks to benefits in the research from the cultural perspective of the potential participants.	0	8.3	91.7	0	0	100	0	0	100	0	4.2	95.8		√
	Time	New: Researchers could spend time building trust with communities in focus and participants to promote engagement with the study.	0	16.6	83.4	0	0	100	0	0	100	0	8.3	91.7		√
Research Stage 3: Measurement	Language	Researchers should consider the use of interpretation and/or translation services.	0	9.1	90.9	0	0	100	0	0	100	0	4.2	95.8	√	
	Language	Researchers could conduct data collection in the language of the participant.	0	25	75	0	0	100	0	0	100	0	16.6	83.4		
	Flexibility	Researchers could evidence that they have considered the resources, for example, time, required to collect the data while respecting cultural preferences/behaviours and accommodations.	0	33.3	66.7	0	20	80	0	0	100	0	25	75		
	Relevance	Researchers need to consider the applicability/validity of existing tools with the communities they work with.	0	16.6	83.4	0	20	80	0	0	100	0	33.3	66.7		
	Contextuality	New: Researchers could consider participants' experiences of culture in relation to how they feel, think, express and behave and how this may influence data being collected in collaboration with appropriate stakeholders.	0	8.3	91.7	0	10	90	0	0	100	0	8.3	91.6		√
	Communication Styles	New: Researchers need to consider how communication is performed with non ‐native English speakers in order to ensure their involvement is facilitated in the research process.	0	8.3	91.7	0	0	100	0	0	100	0	4.2	94.8		√
Research Stage 4: Data Analysis and Interpretation	Language	Researchers should consider the use of interpretation and/or translation services.	8.3	16.7	75	0	0	100	0	0	100	8.3	16.7	75		
	Contextuality	Researchers could seek participants' experiences and expressions of culture in relation to thoughts, feelings and behaviours and how this influences data interpretation.	0	0	100	0	0	100	0	0	100	0	0	100	√	
	Relevance	Researchers could include a strategy of comparator groups to identify cultural similarities and differences.	8.3	16.7	75	0	70	30	50	0	50	8.3	37.5	62.5		
	Empowerment	Researchers could consider including a reflexivity statement on cultural competency.	16.7	33.3	50	10	50	40	0	50	50	12.5	40	47.5		
	Contextuality	Researchers could consider co‐analysis of data together with the community involved.	8.3	8.3	83.4	10	30	60	0	0	100	8.3	12.5	79.2		
	Relevance	New: Researchers could consider including a reflexivity statement on how their own culture and experiences influence the research process they are involved in.	8.3	8.3	83.4	20	40	40	0	50	50	12.5	25	62.5		
	Relevance	NEW: Researchers could consider the applicability of research findings in relation to diverse community groups.	16.6	8.3	75.1	0	10	90	0	50	50	8.3	12.5	79.2		
	Relevance	New: Researchers could consider sense‐checking the findings with communities in focus to ensure the analysis is calibrated to reflect the experiences expressed.	0	8.3	91.7	0	10	90	0	0	100	0	8.3	91.6		√
Research Stage 5: Dissemination	Empowerment	Study findings could be disseminated to levels of involvement (participants to communities) for a range of audiences.	0	33.3	66.7	0	0	100	0	0	100	0	16.7	83.3		
	Relevance	Researchers could consider alternative approaches that acknowledge diversity and culture of sharing study results that have been informed by participants' experiences of thoughts, feelings and behaviours in relation to the studied phenomenon.	0	8.3	91.7	0	0	100	0	0	100	0	4.2	94.8		√
	Reciprocation	Researchers are to disseminate findings in a meaningful way to participants so that the study population benefits from it.	18.1	18.1	63.7	0	7.2	92.8	0	0	100	7.7	3.8	88.4	√	

In Round 2, 32 recommendations and 34 measures were assessed. 13 recommendations and 12 measures achieved consensus. 18 recommendations and 18 measures that did not achieve consensus in either round were initially flagged for removal. Because our aim was to produce a framework pairing each recommendation with a corresponding measure, we retained three measures that—unlike their matched recommendations—had not reached consensus for further discussion at the workshop. Furthermore, six recommendations and six measures that received a median score of 5, although not meeting the predefined consensus threshold, were retained for discussion in the consensus workshop to validate the findings and ensure that no important recommendations and measures were overlooked. A total of 26 recommendations and 26 measures were taken forward to the workshop.

### 6.2 Consensus Workshop

Participants were presented with the survey results and engaged in a focus group discussion on areas of agreement and disagreement (the participants' details can be found in Supplementary File 2). Each recommendation and measure, including those reaching consensus in the survey rounds, was carefully reviewed. Ultimately, 22 recommendations and 22 measures were refined for clarity and precision, resulting in a final set of 25 recommendations and 25 measures that informed the development of a culturally competent research framework (see Supplementary File 4 for further details).

Workshop discussions focused on five research stages across nine culturally competent criteria outlined in Table 1, resulting in final agreement on recommendations and measures that are relevant to each distinct stage. Themes that were discussed in relation to each research stage are described below. The consensus rating and the consensus trajectory of each recommendation and the full list of measures are provided in Supplementary File 3. The measures were not described here as they did not introduce any additional culturally competent principles or guidance beyond those already captured in the developed recommendations.

During the workshop, panellists unanimously agreed to replace ‘could’ with ‘should’ in all recommendations, reinforcing that these practices are integral rather than optional for culturally competent research. As one community lead noted, ‘*Rather than being an option, it should be something we should think about doing’* (CL1).

1. Formulating the Area of Research Focus and Interest

**Figure 3:**
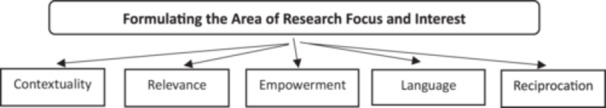
Formulating the area of research focus and interest.

The survey established consensus on recommendations across four criteria: contextuality, relevance, language and empowerment (see Figure 3). Seven recommendations/measures reached consensus during the survey stage, and four achieved consensus based on the workshop. With the workshop addition of reciprocation, the final output included 11 reframed recommendations and measures. Table 5 shows recommendations that reached consensus and contributed to the final framework.

**Table 5:** Recommendations that reached consensus for five criteria when formulating the area of research focus and interest.

Initial recommendation	Final recommendation	Consensus reached		
		S1	S2	Workshop
**Contextuality**				
Researchers could seek to study populations' experiences of culture about how they feel, think, express and behave within the phenomenon/area of interest and how this may underpin their perspectives, expressions and behaviours.	Researchers should explore how diverse populations experience culture—how they feel, think, express themselves, behave and practice daily life—and how these experiences shape their perspectives and actions within the area of interest.		√	√
Researchers could align the area of research focus with research sensitivity that respects the study populations' age, sexual orientation, cultural heritage, social class, work situation and gender inequity.	Researchers should ensure their research focus is sensitive to the community of interest by considering intersecting factors such as age, generation, sexual orientation, cultural heritage, social class, work conditions and gender inequality.	√		√
The researchers could undergo cultural competency training.	Researchers should engage in ongoing cultural humility training that encourages self‐reflection on their attitudes, positionality and openness to cultural differences, alongside cultural competency training focused on the specific community of interest.			√
Researchers need to consider building direct relationships with the community in focus.	Researchers should consider creating capacity within communities of interest.		√	√
**Relevance**				
Researchers could engage with community leaders who can provide culturally sensitive information to support research problem formulation.	Researchers should equally engage with diverse members, leaders and experts in their field within the community in focus who can provide culturally sensitive information to support research problem formulation.		√	√
Researchers could include a patient/public member in the research team to guide culturally sensitive problem formulation.	Researchers should include members of the community/public members in the research team to guide culturally sensitive problem formulation.		√	√
Researchers should have a strategy to capture and monitor the impact and value of patient and public involvement.	No changes made to the initial recommendation.			√
**Language**				
Researchers could enquire about the need for interpretation and/or translation services.	Researchers should enquire about the need for using a variety of translation and interpretation services (such as transcription of interviews or parts of them in the original language, using lay researchers' services and so forth) as well as transcription for people with accessibility needs.	√		√
Researchers could consider the need for additional funding dedicated to covering costs for translation and interpretation.	Researchers should consider the costs and benefits of using a variety of translation and interpretation services (such as transcription of interviews or parts of them in the original language, using lay researchers' services and so forth).		√	√
**Empowerment**				
Researchers could show evidence to demonstrate their appreciation of power dynamics between them and different groups and communities involved.	Researchers should show evidence to demonstrate their appreciation of the power dynamics between them and the different groups and communities involved.			√
**Reciprocation**				
Researchers could ask communities involved in the research what forms of enrichment beyond monetary compensation participants would prefer.	Researchers should work with members of the diverse community of interest to discuss and agree on fair compensation strategies for participants' involvement in the research.			√

All panel groups at the survey and workshop strongly emphasised the need to consider cultural experiences and individual characteristics of minorities and diverse populations. Two recommendations underscored intersectionality, cultural approaches and individuality. Participants stressed that ‘*culture that is always changing and evolving*’ and ‘*it's vital to question established cultural beliefs’* (R1).

Participants advocated shifting from ‘*cultural competency training*’ to ‘*cultural humility training*’ to highlight the need for researcher self‐reflection, continuous learning and openness. This reframing ensures that cultural understanding is not a ‘*tick box*’ (R3) exercise but an ongoing commitment.

Recommendations on translation and interpretation services emerged as an important area of debate. Several participants (CL3, R4 and T/I2) stressed that translation and interpretation were essential for equitable participation, noting that language barriers frequently prevent community members from engaging fully with research processes. However, in a research context, it was highlighted that the term ‘translation’ was interpreted differently across the group (R4). Some referred to translating written materials or providing interpreters, which were viewed as manageable costs, while others pointed out that multilingual transcription was ‘phenomenally expensive’, hence never used (R4 and T/I2). Participants described how, in practice, high transcription costs often result in selective translation of only key ‘nuggets’, meaning that a significant part of data from non‐English interviews may never be fully captured or used (R4). These discussions revealed differing assumptions and real‐life examples of linguistic accommodation and suggested a flexible, diverse yet realistic approach to the types of translation and interpretation capacity needed in culturally inclusive research.

Also, the discussion revealed that language‐focused approaches to research may unintentionally obscure cultural differences within linguistic groups. One interpreter (CL2) emphasised that *‘within Spanish, there are different cultures … different meanings of things’,* noting that a single translation may fail to capture important nuances. This prompted broader debate about how cultural heterogeneity should be reflected in research materials.

The survey strongly supported including patient/public members in research teams for culturally sensitive problem formulation. Recommendations for monitoring public involvement impact and building community relationships also garnered strong support (91.7%). Workshop experts addressed power imbalances, reframing ‘*relationship*’ as ‘*creating capacity within communities*’ (CL1), prioritising empowerment over extraction. A community leader (CL4) described this engagement as ‘*building bridges*’ between people and researchers, which is ‘*empowering in nature*’.

Expanding on this, the workshop emphasised the need to broaden engagement beyond community leaders to include community members and subject matter experts, ensuring diverse perspectives and addressing concerns about solely relying on leaders who may have health literacy limitations or not be representative. The panellists argued for wider community participation during the research problem formulation stage. A researcher (R3) stressed reflexivity: ‘*this isn't just in a kind of cynical way, but just being reflexive throughout this process of engaging community leaders. Because not everybody is on the same footing. And not everybody may have an understanding of research either. So good to kind of reflect on the process’*.

Beyond Meleis' original framework, study findings suggested including ‘*language*’ as a criterion to consider. Workshop participants (R4, CL1 and T/L2) emphasised the importance of ‘*precision in language nuance*’ and ‘*diversity of transcription options to address accessibility needs related to translation and interpretation services*’ for culturally competent research.

2. Recruitment: Ensuring Inclusive and Ethical Engagement of Culturally Diverse Participants

**Figure 4:**
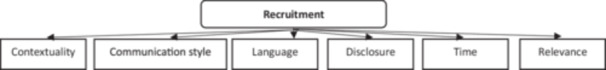
Recruitment culturally competent criteria.

In the recruitment stage, six criteria achieved a strong consensus (see Figure 4). Five recommendations were agreed upon in both survey rounds and workshop, with one additional recommendation under the relevance criterion ‘Have the researchers demonstrated how their recruitment strategy has facilitated a diverse insight of the study population?’ added at the workshop. Table 6 shows recommendations that reached consensus and contribute to the final framework.

**Table 6:** Recommendations that reached consensus for six criteria during recruitment.

Initial recommendation	Final recommendation	Consensus reached		
		S1	S2	Workshop
**Contextuality**				
Researchers could engage with a trusted/respected person within their field/community who can provide culturally sensitive information to support study recruitment.	Researchers should engage with a group of trusted/respected people within the community of interest who can provide culturally sensitive information to support study recruitment.		√	√
**Communication style**				
Researchers could develop a communication strategy tailored to community involvement.	Researchers should develop a tailored communication strategy with the community of interest.	√		√
**Language**				
Researchers could consider the use of interpretation and/or translation services.	Researchers should consider the costs and benefits of using a variety of translation and interpretation services (such as transcription of interviews or parts of them in the original language, using lay researchers' services and so forth).	√		√
**Disclosure**				
Ethics should be discussed through the lens of ethnicity and culture, including the provision of culturally appropriate study explanations, assessing the cultural impediments to what researchers consider truly informed consent or the degree to which it is achieved, considering the ratio of risks to benefits in the research from the cultural perspective of the potential participants.	Ethics should be addressed through the lens of ethnicity and culture by: (i) ensuring ethics committees receive cultural training, (ii) providing culturally appropriate explanations of the study, (iii) assessing cultural barriers to achieving truly informed consent and (iv) evaluating the risk–benefit ratio from the cultural perspective of potential participants.		√	√
**Time**				
Researchers could spend time building trust with communities in focus and participants to promote engagement with the study.	The same.		√	√
**Relevance**				
Researchers should demonstrate how they have tailored recruitment and facilitated a diverse representation of the community of interest.	The same.			√

There was unanimous agreement that researchers should engage trusted individuals within the community or field to facilitate culturally appropriate and respectful recruitment.

The use of interpretation and/or translation services was widely supported to improve accessibility and inclusivity.

Ethical procedures during research created a debate among panellists during the workshop, particularly when exploring how informed consent is understood and communicated. Some (CL2 and T/I2) emphasised that cultural norms around consent differ across groups and that study information must be explained in ways that reflect these differences. In contrast, several researchers (R4 and R6) argued that the primary barrier lies within ethics review processes rather than within communities. They noted that ethics committees frequently require lengthy, complex consent documents that undermine accessibility, even when researchers advocate for clearer and culturally appropriate materials. Participants also highlighted the difficulty of wording ethics‐related guidance in a way that ‘gets past the Ethics Committee’ (R6) that is acceptable to ethics committees while still reflecting diverse cultural perspectives.

The communication style was also highlighted as an important criterion, where the key was to consider various forms of communication, including nonverbal ones, which would allow enough flexibility for participants when designing and conducting research.

3. Measurement: Culturally Valid and Accessible Data Collection

**Figure 5:**
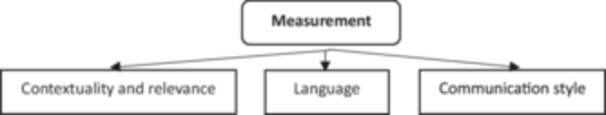
Measurement of culturally competent criteria.

In the measurement stage, three criteria reached consensus with three recommendations (see Figure 5). Table 7 shows recommendations that reached consensus and contribute to the final framework.

**Table 7:** Recommendations that reached consensus for three criteria during measurement.

Initial recommendation	Final recommendation	Consensus reached		
		S1	S2	Workshop
**Contextuality**				
Researchers could consider participants' experiences of culture in relation to how they feel, think, express and behave and how this may influence data being collected.	Researchers should consider participants' experiences of culture in relation to how they feel, think, express and behave and how this may influence data being collected in collaboration with appropriate stakeholders.		√	√
**Language**				
Researchers should consider the use of interpretation and/or translation services.	Researchers should consider the costs and benefits of using a variety of translation and interpretation services (such as transcription of interviews or parts of them in the original language, using lay researchers' services and so forth).	√		√
**Communication style**				
Researchers need to consider how communication is performed with non ‐native English speakers in order to ensure their involvement is facilitated in the research process.	Researchers should consider how they engage with non‐native speakers to ensure their involvement is facilitated in the research process.		√	√

There was 100% agreement that researchers should attend to how participants' cultural experiences—shaped by how they feel, think, express and behave—can influence the data being collected.

The provision of interpretation and/or translation services was seen as vital for ensuring accurate and inclusive data from participants who may not speak the dominant research language.

Researchers were advised to attend carefully to how communication is conducted with a person who did not speak English, ensuring that their engagement in the research process is meaningful and not compromised by language barriers.

Panellists strongly supported diverse translation and interpretation strategies (e.g., lay researchers and original language transcription), stressing cost–benefit assessment. These recommendations and measures, reaching initial consensus in Round 1, were refined for clarity. Panellists emphasised that effective interpretation and translation are core to inclusive research. As a policymaker (PM1) noted, ‘*So it's not something you kind of could do; it's something you should be thinking about: have I fulfilled the needs of participants*?’

4. Data Analysis and Interpretation: Culturally Reflexive and Collaborative Interpretation of Findings

**Figure 6:**
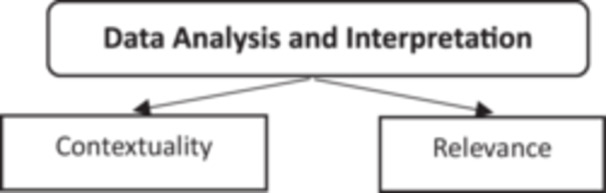
Data analysis of culturally competent criteria.

In the data analysis and interpretation stage, two criteria reached consensus (see Figure 6). Table 8 shows recommendations that reached consensus and contribute to the final framework.

**Table 8:** Recommendations that reached consensus for two criteria during data analysis.

Initial recommendation	Final recommendation	Consensus reached		
		S1	S2	Workshop
**Contextuality**				
Researchers could seek participants' experiences and expressions of culture in relation to thoughts, feelings and behaviours and how this influences data interpretation.	Researchers should explore how diverse populations experience culture—how they feel, think, express themselves, behave and carry out daily practices—and how these experiences shape their perspectives and actions within the area of interest.	√		√
**Relevance**				
Researchers could consider sense‐checking the findings with communities in focus to ensure the analysis is calibrated to reflect the experiences expressed.	Researchers should consider co‐analysis of data together with the community of interest and reimburse them for their time and input.		√	√

Panellists emphasised that cultural expressions could shape data interpretation. This recommendation (92% agreement) and its measure (100% agreement) confirmed inclusive analysis as a core competency. As a community leader stated, ‘*Get feedback from the people who are in distress and a proper representation of their unlimited experiences*’ (CL4). Culture, however, is ‘*a lot more than feelings [and] thoughts…. It's a lot to do with practices as well. It's not just what people say, it's what people do*’ (R3).

While survey rounds did not achieve consensus, the panel added co‐analysis with community members as a recommendation and indicator in the workshop. This acknowledged the value of collaborative interpretation, especially in participatory research. Co‐analysis requires funding for community members' time and ‘*a lot of longstanding trust and rapport with your participants*’ (R3).

5. Dissemination: Sharing Study Results in a Culturally Informed, Empowering and Accessible Manner

**Figure 7:**
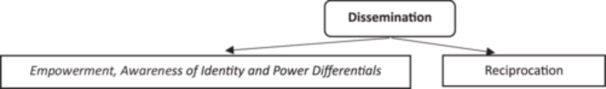
Dissemination of culturally competent criteria.

In the dissemination stage, two criteria, including empowerment, awareness of identity and power differentials and reciprocation reached consensus (see Figure 7). Table 9 shows recommendations that reached consensus and contribute to the final framework.

**Table 9:** Recommendations that reached consensus for three criteria during dissemination.

Initial recommendation	Final recommendation	Consensus reached		
		S1	S2	Workshop
**Empowerment, Awareness of Identity and Power Differentials**				
Researchers could consider alternative approaches that acknowledge diversity and the culture of sharing study results that have been informed by participants' experiences of thoughts, feelings and behaviours in relation to the studied phenomenon.	Researchers should explore and report on how diverse populations experience culture—how they feel, think, express themselves, behave and carry out daily practices—and how these experiences shape their perspectives and actions within the area of interest.		√	√
Study findings could be disseminated to levels of involvement (participants to communities) at a range of audiences.	Study findings should be shared with all levels of involvement—from individual participants to the broader community of interest—as well as with diverse audiences.			√
**Reciprocation**				
Researchers need to disseminate findings in a meaningful way to participants so that the study population benefits from it.	Researchers should disseminate findings in an understandable, meaningful way to participants so that the study population benefits from it and the research results work for the community of interest.	√		√

Panellists agreed on three recommendations and measures for the dissemination stage: acknowledging diversity and sharing participant‐informed results. This stage is critical for providing meaningful research results to participants. Cultural training often lacks practical application, emphasising the need to know what works for the community, not just the researcher.

## 7 Discussion

This study aligns with recent scholarship in its aim to advance culturally competent, diversifying approaches to research. This work moves beyond static checklists, towards a dynamic, multistage framework informed by evidence and co‐designed/co‐developed with expert contributors [62, 63]. This study also contributes to conceptual clarity by positioning the adapted Meleis' framework in relation to culturally competent research. While frameworks such as Campinha‐Bacote's Process of Cultural Competence, the Purnell and Papadopoulos–Tilki–Taylor models, the NIHR Race Equality Framework, and the INCLUDE Ethnicity Framework each offer useful principles, they primarily focus on clinical care, education or organisational self‐assessment and therefore do not provide the applicable, ready‐to‐use guidance required for the research process. These models informed our initial scoping and enhanced the need for application clarity, but were not suitable for direct use as they were not designed to support iterative decision‐making across the research lifecycle. In contrast, our findings extend Meleis' original criteria by translating them into practical recommendations and measurable indicators co‐developed with researchers, community representatives, translators and policy stakeholders. This results in a more actionable and adaptable framework, which is not restricted to health research but is transferable to other disciplines. Using a modified Delphi method, the final framework included 25 co‐developed recommendations and 25 implementation measures.

A key finding was the centrality of the ‘contextuality and relevance’ criteria, which were identified as critical across four research stages (from problem formulation and conceptualisation, through to data analysis). This echoes earlier research that advocates moving beyond checklists of competence [64] and instead embedding an understanding of culture as a dynamic process throughout the research lifecycle [65]. In addition, the criteria of ‘participant empowerment and reciprocation’ emerged as strong enablers of inclusive research when prioritised during research design and dissemination—specifically, our findings suggest that these criteria are often underutilised at early and late research stages, despite their importance for legitimacy and impact. By anchoring participant empowerment in concrete decisions about representation and knowledge ownership, this work challenges tokenistic or performative inclusion. This aligns with existing evidence on centring the voices of populations in focus across the research process, while challenging the conventional notion of the researcher as ‘expert’ [66]. Following the argument on the complementary nature of cultural competence and PPIE, our findings suggest that empowerment and reciprocation act as mechanisms that can strengthen PPIE but also extend beyond it. Experts highlighted power imbalances and reframed ‘relationship’ as ‘creating capacity within communities’, describing genuine engagement as ‘empowering in nature’ rather than extractive. They also emphasised the importance of involving wider participation from community members and subject‐matter experts. These insights suggest that while PPIE focuses on who is involved in shaping research, the framework focuses on how to attend to cultural equity and knowledge ownership even in studies where formal PPIE structures are limited. Language also emerged as a critical theme, with participants calling for inclusive and diverse communication strategies to be included within research plans. Participants broadly agreed that translation and interpretation were essential for equitable participation, yet differed in how they understood and operationalised those needs. Community leader participants emphasised language barriers as an obstacle to inclusion, whereas researchers highlighted that translation could refer to very different practices, from translating written materials to providing interpreters to producing full multilingual transcripts with substantial variation in cost, feasibility and risks associated. Often, the pragmatic reasons of high transcription costs may lead to selective translation and even loss of valuable data. The workshop enabled the participants to discuss disagreement in different formats (written and verbal), where the need for realistic and flexible translating approaches was agreed on.

This contributes to the existing debate on how interpreters must adapt to culturally and linguistically diverse contexts, especially in community, medical and political settings [67, 68]. It is argued that no single translation or interpretation strategy fits all contexts, reinforcing the need for plural and inclusive communication mechanisms [69]. However, our findings extend this by emphasising not only the need for translation and interpretation, but also the funding and infrastructure required to implement it meaningfully. Translation and interpretation, participants argued, must be embedded, not treated as last‐minute logistical add‐ons. In this way, this study helps articulate how institutional planning processes either enable or constrain culturally competent practice.

Communication style is another critical dimension of culturally competent research. Participants highlighted the importance of developing flexible communication strategies that would be tailored to the community's needs, including recognition of and valuing multiple forms of communication, such as verbal and nonverbal approaches, that may offer participants more comfortable and meaningful ways to engage and, most importantly, reduce barriers to participation. This aligns with existing guidance such as the U.S. National CLAS Standards, which call for linguistically and culturally appropriate communication and the use of modified, alternative or multimodal methods to support equitable engagement and minimise participation barriers [11].

Power dynamics within community engagement were another central concern. While much of the previous literature has acknowledged the importance of stakeholder involvement [70], participants in this study went further by arguing that over‐reliance on designated ‘community leads’ risks reproducing existing hierarchies—instead, they advocated for broader inclusion strategies that reflect a diversity of lived experience, aligning with recent work on epistemic justice in research [71]. This reframing of who is considered a legitimate contributor challenges researchers to rethink their engagement strategies and attend to hidden gatekeeping structures.

The call for continuous researcher training reinforced existing critiques of ‘cultural competence’ as a static skillset. Participants strongly endorsed a shift towards cultural humility, consistent with emerging frameworks that emphasise reflexivity, positionality and relational accountability [72]. Our findings offer practical recommendations for operationalising this approach across the entire research process, suggesting that training should not be an isolated event but an iterative, embedded practice.

Finally, a major shift in framing occurred during the consensus workshop: recommendations initially framed as optional (‘could’) were reframed as compulsory (‘should’), reflecting a collective agreement that culturally competent research should be the default standard—not a discretionary application. This strengthened language aligns with the growing expectations of many research funders, who now require applicants to explicitly address cultural competence, inclusion and equity in their grant proposals [73]. By embedding these practices as a baseline standard, the culturally competent research framework supports researchers in meeting both ethical imperatives and funding requirements. This study has advanced the evidence base by integrating CBPR and cultural humility principles [24, 74], addressing critiques that cultural competence is too often conceptualised in static or decontextualised ways [75, 76]. Rather than offering a generic checklist, this framework was co‐developed through an iterative, multi‐stakeholder process, making it adaptable, nuanced and responsive to real‐world research challenges.

## 8 Implications for Policy and Practice

This framework offers practical, ready‐to‐use guidance for researchers, professionals, funders and institutions seeking to embed cultural competence meaningfully within research. Each recommendation is accompanied by a measurable indicator, supporting monitoring, accountability and continuous improvement. This has immediate utility for researchers, ethics committees and grant reviewers, conducting research or developing training or frameworks.

A strong consensus on the compulsory use of the framework reflects that culturally competent practices should be institutional norms, not aspirational ideals. Although developed primarily for researchers, there are opportunities for the recommendations to inform practice in other areas, including policy development, funding criteria, ethics review processes and organisational training.

## 9 Strengths and Limitations

A key strength of this study is its inclusive, co‐productive approach, involving community leaders, researchers, policymakers and translators throughout the research process. The modified Delphi method facilitated structured consensus while allowing space for critical discussion, ensuring that findings were informed by evidence, theory and practice.

However, there are limitations. While participants offered diverse professional and community insights, a broader sampling could offer further opinions and perspectives with added breadth of diversity and context in mind. As such, the expert consensus generated through this Delphi process reflects professional, policy, academic and community‐facing perspectives rather than direct lived experience input from patients or carers. This was a deliberate methodological choice aligned with the study's focus on producing high‐level, cross‐cutting guidance for culturally competent research practice, rather than context‐specific recommendations tied to particular conditions or populations. Additionally, consensus thresholds may have excluded valuable minority perspectives, underscoring the need to complement consensus methods with qualitative approaches to capture complexity and dissent.

## 10 Future Research

Future research should test the usability, implementation and impact of the framework in different research contexts. There is also a need to explore how researchers and professionals adopt and integrate the framework into ethics governance, funding criteria and researcher training programmes. Finally, future work should explore digital and language equity in more depth, particularly as technology increasingly mediates recruitment, participation and dissemination.

## 11 Conclusion

This study contributes a dynamic, evidence‐informed framework for culturally competent research developed through a rigorous, multi‐stakeholder modified Delphi process. Moving beyond static checklists, this framework centres contextuality and relevance across multiple research stages, emphasising culture as a fluid, embedded process rather than a fixed attribute. Key contributions include highlighting empowerment and reciprocation as critical yet underused enablers of inclusive research, advancing plural and funded language strategies, and challenging traditional power dynamics in community engagement. By framing cultural competence as an iterative practice rooted in humility, reflexivity and relational accountability, the framework addresses longstanding critiques of static competence models. Importantly, consensus shifted recommendations from optional to compulsory, underscoring the imperative that culturally competent research be the default standard. Now available as an accessible online resource with measurable indicators, this culturally competent research framework offers practical application for researchers, institutions, funders and ethics bodies to embed cultural competence systematically—towards more equitable, inclusive and accountable research.

## 12 Additional Information

This framework has been created as an online toolkit to support anyone who is involved in any stage of the research cycle, that is, the formulation of research problems (conception), the recruitment of participants, the measurement, analysis and interpretation of findings, and the reporting and dissemination. The CCRF toolkit is designed to support researchers (e.g., based in research centres and Universities) and people involved in conducting research that could influence the design and development of health and care services, including stakeholders working in public and policy organisations (e.g., local authorities and charities). It is available here with a supporting worksheet: Culturally Competent Research Framework.

## Author Contributions

**Evgenia Stepanova:** investigation, methodology, validation, visualisation, writing – review and editing, writing – original draft, data curation, resources, conceptualisation, formal analysis, software. **Matthew Cooper:** conceptualisation, writing – review and editing, validation, project administration, formal analysis, supervision. **Anna Robinson‐Barella:** writing – review and editing, validation, conceptualisation. **Vicki Harris:** conceptualisation, writing – review and editing. **Abbie Rance:** conceptualisation. **Abbie Husband:** conceptualisation, funding acquisition, writing – review and editing. **David Wright:** funding acquisition, writing – review and editing, conceptualisation. **Fabi Lorencatto:** funding acquisition, writing – review and editing, conceptualisation. **Clare Tolley:** funding acquisition, writing – review and editing. **Wade Tovey:** funding acquisition, conceptualisation. **Geoff Wong:** conceptualisation, funding acquisition, writing – review and editing. **Daniel Cowie:** conceptualisation, funding acquisition, writing – review and editing. **Kerry Parker:** conceptualisation. **Elise Crayton:** writing – review and editing, funding acquisition, conceptualisation. **Hamde Nazar:** conceptualisation, investigation, funding acquisition, writing – review and editing, visualisation, validation, methodology, project administration, supervision, formal analysis, resources.

## Disclosure

The funders had and did not have a role in study design, data collection and analysis, decision to publish, or preparation of the manuscript.

## Ethics Statement

Ethical approval (Ref: 51189/2023) was granted by the Ethics Committee at the Faculty of Medical Sciences, Newcastle University. Data were collected with electronic informed consent of participants and stored securely on the university's server.

## Consent

A consent statement was included on each survey's introductory page. Participants were required to complete the consent statement prior to completing the survey.

## Conflicts of Interest

The authors declare no conflicts of interest.

## Supporting information


**Figure 1:** A flow diagram of the study selection process. **Table 1:** List of excluded papers (n=40) with reason for exclusion. **Table 2:** Developing data sources: a rapid review and study team synthesis.

**Table 1:** Coding details of workshop panellists.

**Table 1:** Process of reaching consensus across modified Delph.

**Supporting File 4:** The final framework.

## Data Availability

The data that support the findings of this study are available on request from the corresponding author. The data are not publicly available due to privacy or ethical restrictions.
