# Supplementary material for: Cultural Competency in Research: A Practical Framework for Use by Researchers, Policymakers, Community Leads and Others When Working With People From Diverse Groups
Source: Health Expect. 2026 Jan 13;29(1):e70544. doi: 10.1111/hex.70544 (PMC12796843; doi:10.1111/hex.70544)
Supplement: Supplementary file 1 — Figure 1: A flow diagram of the study selection process. Table 1: List of excluded papers (n=40) with reason for exclusion. Table 2: Developing data sources: a rapid review and study team synthesis. [file HEX-29-e70544-s004.docx]

Supplementary File 1: Process of rapid review and research team synthesis

**Search Strategy and Eligibility Criteria**

We systematically searched five databases—PsycINFO, ERIC, PubMed, Web of Science Core Collection, and Google Scholar—for English-language publications from 1996 to 2024. Included studies explicitly applied or discussed Meleis’ cultural competence criteria in research contexts. Exclusions were studies focusing solely on educational settings, lacking reference to Meleis’ framework, non-English publications, or insufficient methodological detail.

**Screening and Selection Process**

One reviewer (ES) screened all titles and abstracts, followed by full-text reviews. Discrepancies were resolved through discussion with a second and third reviewer (HN and MC).

**Data Extraction and Piloting**

A standardised data extraction form was developed and piloted on three diverse studies to ensure clarity and consistency. Extracted data included: research setting, population focus, study design, implementation and application of each Meleis criterion across stages of the research cycle and how the application of the criteria was measured (if at all).

**Results**

A total of 352 records were screened and 53 were assessed potentially eligible based on title and abstract. Of these, eight records met the inclusion criteria, spanning the disciplines of nursing, social work, and health research. The studies focused on diverse populations, including Korean women, language minorities, and marginalised communities. The publications were from the late 1990s to early 2000s, reflecting an early engagement with cultural competence in health-related research. All included records were conducted in or affiliated with institutions in the United States, with some focusing on immigrant or minority communities originating from East Asia and Latin America.

A flow diagram of the study selection process is shown in Figure 1, in line with PRISMA guidance^[[1]](#footnote-1)^.


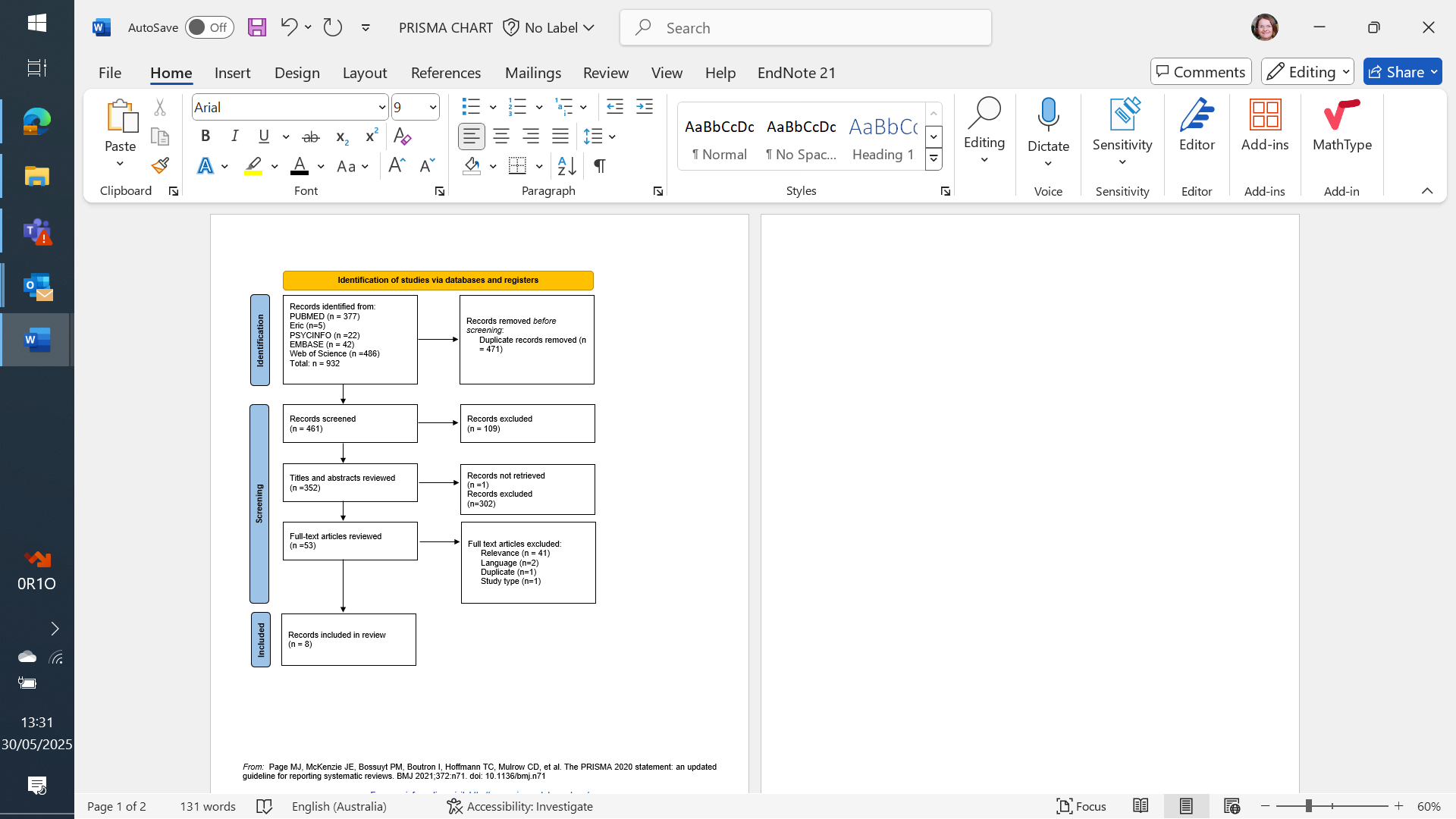


Figure 1: A flow diagram of the study selection process

The included studies applied Meleis’ cultural competence criteria in varied ways. Five studies focused on culturally competent scholarship (Jacobson et al.,2005; Meleis, 1999; Meleis, 2001; Meleis and Im., 1999; Mendias and Guevara, 2021), one study explored the application of the framework in social work (Casado et *al*., 2012) while two others offered development and validation of the Meleis’ framework (1996) (Im et *al*., 1999a, Im et *al.,* 1999b). The rapid review identified recommendations and/or measures in which Meleis’ (1996) cultural competence criteria have been used across five stages of the research cycle.

The eight included studies, primarily situated in nursing and social work disciplines in the United States, demonstrated a range of recommendations that contribute to culturally competent research practice.

**Table 1: List of excluded papers (n=40) with reason for exclusion**

| **No** | Excluded paper | Reason for exclusion |
| --- | --- | --- |
| **1** | Bhutta ZA, Chen L, Cohen J, Crisp N, Evans T, Fineberg H, Frenk J, Garcia P, Horton R, Ke Y, Kelley P, Kistnasamy B, Meleis A, Naylor D, Pablos-Mendez A, Reddy S, Scrimshaw S, Sepulveda J, Serwadda D, Zurayk H. Education of health professionals for the 21st century: a global independent Commission. Lancet. 2010 Apr 3;375(9721):1137-8. doi: 10.1016/S0140-6736(10)60450-3. PMID: 20362799. | Commentary, no evidence of Meleis cultural competence framework |
| **2** | Carter M, Hathaway D, Johnson J, Mundinger M. Response to Meleis's article. Nurs Outlook. 2016 Sep-Oct;64(5):408. doi: 10.1016/j.outlook.2016.06.003. Epub 2016 Jun 23. PMID: 27445087. | Response, no evidence of Meleis cultural competence framework |
| **3** | Castner J, Amiri A, Rodriguez J, Huntington-Moskos L, Thompson LM, Zhao S, Polivka B. Advancing the symptom science model with environmental health. Public Health Nurs. 2019 Sep;36(5):716-725. doi: 10.1111/phn.12641. Epub 2019 Jul 16. PMID: 31310379; PMCID: PMC6855393. | no evidence of Meleis cultural competence framework |
| **4** | Palmer MA. Transforming urban environmentsIf the Past Teaches, What Does the Future Learn?John T. Murphy and Carole L. Crumley, Eds. TU Delft Publishing, 2022. 124 pp. Science. 2022 Aug 19;377(6608):823. doi: 10.1126/science.ade1308. Epub 2022 Aug 18. PMID: 35981023. | no evidence of Meleis cultural competence framework |
| **5** | Cuff P, Meleis A, Cohen J. Institute of Medicine's Global Forum on Innovation in Health Professional Education. Neurology. 2014 Feb 25;82(8):713-5. doi: 10.1212/WNL.0000000000000142. PMID: 24566224 | no evidence of Meleis cultural competence framework |
| **6** | Davidson PM, Meleis AI. Sex and gender matters: The health of women and girls determines the health of our modern world. Health Care Women Int. 2016 Aug;37(8):817. doi: 10.1080/07399332.2016.1218720. PMID: 27485885. | Commentary, news, no evidence of Meleis cultural competence framework |
| **7** | Dhas BN, Wagman P. Occupational balance from a clinical perspective. Scand J Occup Ther. 2022 Jul;29(5):373-379. doi: 10.1080/11038128.2020.1865450. Epub 2020 Dec 31. PMID: 33382004 | The focus on The Meleis' Theory to Practice to Theory strategy |
| **8** | Meanings and social representations attributed to children's fears: promotion for positive parenting  R. Fernandes, A. Mendes, J. Ferreira, F. Neto, A. Navarro and G. Marques | Spanish language |
| **9** | [Health professionals for a new century: transforming education to strengthen health systems in an interdependent world]  J. Frenk, L. Chen, Z. A. Bhutta, J. Cohen, N. Crisp, T. Evans, et al.  Rev Peru Med Exp Salud Publica 2011 Vol. 28 Issue 2 Pages 337-41 | Spanish language |
| **10** | Frimpong, Samuel, Riza Yosia Sunindijo, Cynthia Changxin Wang, Elijah Frimpong Boadu, and Ayirebi Dansoh. 2023. "A Conceptual Framework to Promote the Transition to Positive Mental Health among Young Construction Workers" Buildings 13, no. 4: 1025. https://doi.org/10.3390/buildings13041025 | Reference to Transition Theory, no evidence of Meleis cultural competence framework |
| **11** | Cultural competency in social work scholarship and research on Latinos in the United States: A content analysis of peer-reviewed social work journals  N. M. Hadley | Conference paper, no evidence of Meleis cultural competence framework |
| **12** | Marianne Hattar-Pollara, Afaf Ibrah (2000) ‘A STUDY OF THE SPOUSAL ROLE OF EGYPTIAN WOMEN IN CLERICAL JOBS’, Health care for women international., 21(4), pp. 305–317. | no evidence of Meleis cultural competence framework |
| **13** | Hattar-Pollara M, Meleis AI, Nagib H. Multiple role stress and patterns of coping of Egyptian women in clerical jobs. J Transcult Nurs. 2003 Apr;14(2):125-33. doi: 10.1177/1043659602250633. PMID: 12772621. | No evidence of Meleis cultural competence framework |
| **14** | Im EO, Meleis AI. A situation-specific theory of Korean immigrant women's menopausal transition. Image J Nurs Sch. 1999;31(4):333-8. doi: 10.1111/j.1547-5069.1999.tb00513.x. PMID: 10628099. | No evidence of Meleis cultural competence framework |
| **15** | Im E-O, Meleis AI. Meanings of Menopause to Korean Immigrant Women. Western Journal of Nursing Research. 2000;22(1):84-102. doi:10.1177/019394590002200107 | No evidence of Meleis cultural competence framework |
| **16** | Im EO, Meleis AI. An international imperative for gender-sensitive theories in women's health. J Nurs Scholarsh. 2001;33(4):309-14. doi: 10.1111/j.1547-5069.2001.00309.x. PMID: 11775299. | No evidence of Meleis cultural competence framework |
| **17** | Conceptualizing resilience in women older than 85: overcoming adversity from illness or loss B. S. Felten and J. M. Hall J Gerontol Nurs 2001 Vol. 27 Issue 11 Pages 46-53 | Reference to another Meleis framework from 1997, No evidence of Meleis cultural competence framework |
| **18** | Symptom experience during menopausal transition: low income Korean immigrant women  E. O. Im, A. I. Meleis and K. A. Lee  Women Health 1999 Vol. 29 Issue 2 Pages 53-67 | No evidence of Meleis cultural competence framework |
| **19** | Jepsen, K., Lindström, V., Wihlborg, J. *et al.* Newly employed nurses’ transition into their new role in the ambulance service– a qualitative study. *BMC Nurs* 23, 93 (2024). https://doi.org/10.1186/s12912-024-01745-y | No evidence of Meleis cultural competence framework |
| **20** | Caregiving between two cultures: an integrative experience  P. S. Jones, X. E. Zhang, K. Jaceldo-Siegl and A. I. Meleis  J Transcult Nurs 2002 Vol. 13 Issue 3 Pages 202-9 | No evidence of Meleis cultural competence framework |
| **21** | Transforming vulnerability  P. S. Jones, X. E. Zhang and A. I. Meleis  West J Nurs Res 2003 Vol. 25 Issue 7 Pages 835-53 | No evidence of Meleis cultural competence framework |
| **22** | Martins H, Caldeira S. Disclosing and discussing the role of spirituality in the transition theory of Afaf Meleis. Nurs Philos. 2021 Apr;22(2):e12348. doi: 10.1111/nup.12348. Epub 2021 Feb 10. PMID: 33565709. | No evidence of Meleis cultural competence framework |
| **23** | Meleis, A. (2001). SMALL STEPS AND GIANT HOPES: VIOLENCE ON WOMEN IS MORE THAN WIFE BATTERING. Health Care for Women International, 22(4), 313–315. https://doi.org/10.1080/07399330117645 | Editorial, no evidence of Meleis cultural competence framework |
| **24** | Whither international research?  A. Meleis  JOURNAL OF NURSING SCHOLARSHIP 2002 Vol. 34 Issue 1 Pages 4-5 | No evidence of Meleis cultural competence framework |
| **25** | Afaf I. Meleis,  Immigrant transitions and health care: an action plan,  Nursing Outlook,  Volume 45, Issue 1,  1997,  Page 42, | No evidence of Meleis cultural competence framework |
| **26** | Special section on cultural competency  A. I. Meleis  J Prof Nurs 2008 Vol. 24 Issue 3 Pages 131-2 | No reference to Meleis cultural competence framework |
| **27** | Meleis, A. I. (2009). Editorial—ICOWHI Special Issue. *Health Care for Women International*, *30*(3), 181–183. https://doi.org/10.1080/07399330802661988 | No reference to Meleis cultural competence framework |
| **28** | Meleis, A. I. (2009). Editorial—ICOWHI Special Issue. *Health Care for Women International*, *30*(3), 181–183. https://doi.org/10.1080/07399330802661988 | Duplicate |
| **29** | Meleis AI. My wish for a global research agenda in nursing. Rev Lat Am Enfermagem. 2015 Jul-Aug;23(4):569-70. doi: 10.1590/0104-1169.0000.2589. PMID: 26444156; PMCID: PMC4623717. | No reference to Meleis cultural competence framework |
| **30** | Langer A, Meleis AI. News From the International Council on Women's Health Issues. Health Care Women Int. 2015;36(6):635-6. doi: 10.1080/07399332.2015.1048103. PMID: 25985350. | No reference to Meleis cultural competence framework |
| **31** | Meléis, A.I., & Glickman, C.G. (2014). A Passion in Nursing for Justice : Toward Global Health Equity. | No reference to Meleis cultural competence framework |
| **32** | Missal B. Gulf Arab women's transition to motherhood. J Cult Divers. 2013 Winter;20(4):170-6. PMID: 24575592. | No reference to Meleis cultural competence framework |
| **33** | Piper S. Qualitative theory testing as mixed-method research. Journal of Research in Nursing. 2006;11(3):183-193. doi:10.1177/1744987106064633 | No reference to Meleis cultural competence framework |
| **34** | Risk for Complicated Immigration Transition: New Diagnosis for NANDA-International  R. Rifa-Ros, C. Espinosa Fresnedo, M. N. S. Rn, M. Alcazar Paris, B. Rn, L. Raigal Aran and C. Ferre Grau  2019 Vol. 30 Issue 2 Pages 68-72 | No reference to Meleis cultural competence framework |
| **35** | Bridging nursing practice and education through a strategic global partnership  M. Stringer, L. Rajeswaran, K. Dithole, L. Hoke, P. Mampane, S. Sebopelo, et al.  INTERNATIONAL JOURNAL OF NURSING PRACTICE 2016 Vol. 22 Issue 1 Pages 43-52 | No specific reference to Meleis cultural competence framework |
| **36** | Zanotti R. Overcoming national and cultural differences within collaborative international nursing research. West J Nurs Res. 1996 Feb;18(1):6-11. doi: 10.1177/019394599601800101. PMID: 8686291. | No specific reference to Meleis cultural competence framework |
| **37** | Meleis AI, Douglas MK, Eribes C, Shih F, Messias DK. Employed Mexican women as mothers and partners: valued, empowered and overloaded. J Adv Nurs. 1996 Jan;23(1):82-90. doi: 10.1111/j.1365-2648.1996.tb03139.x. PMID: 8708228. | No specific reference to Meleis cultural competence framework |
| **38** | Georges JM. Bio-power, Agamben, and emerging nursing knowledge. ANS Adv Nurs Sci. 2008 Jan-Mar;31(1):4-12. doi: 10.1097/01.ANS.0000311525.50693.9c. PMID: 20531265. | No specific reference to Meleis cultural competence framework |
| **39** | Clark C, Robinson T. Multiculturalism as a concept in nursing. J Natl Black Nurses Assoc. 2000 Dec;11(2):39-43. PMID: 11854988. | No specific reference to Meleis cultural competence framework |
| **40** | Meleis AI. ReVisions in knowledge development: a passion for substance. Sch Inq Nurs Pract. 1998 Spring;12(1):65-77; discussion 79-94. PMID: 9805472. | No specific reference to Meleis cultural competence framework |
| **41** | Marginalization revisited: critical, postmodern, and liberation perspectives  J. M. Hall | No specific reference to Meleis cultural competence framework |
| **42** | Meleis AI. Scholarship and the RO1. J Nurs Scholarsh. 2001;33(2):104-5. PMID: 11439934. | No specific reference to Meleis cultural competence framework |
| **43** | "Opportunities in preparing global leaders in nursing  R. Carty, E. T. O'Grady, O. A. Wichaikhum and J. Bull | Cultural competence criteria is applied to development of educational programmes |
| **44** | Gunaratnam Y. The benefits and challenges of voluntary-academic research partnerships: A critical reflection rooted in Meleis criteria for cultural competent research. Journal of Research in Nursing. 2006;11(6):541-542. doi:10.1177/1744987106065681 | Review, no description on Meleis cultural competency criteria |

**Table 2: Developing data sources: a rapid review and study team synthesis**

| **Research Stage** | **Cultural Competence Criterion** | **Representative Study** | **Rapid review coding** | **Data synthesis:**  **Suggested Recommendations developed from research team synthesis** | **Data synthesis:**  **Suggested Measures developed from research team synthesis** |
| --- | --- | --- | --- | --- | --- |
| **1. Problem Formulation** | Contextuality  Relevance  Reciprocation  Empowerment | Casado et al. (2012) | 1. It is important to consider knowledge where the target population lives and a consideration of environmental factors associated with the problems faced by the study population;  2. The literature review will not be enough to develop contextually relevant research questions  3. Focus groups with community leaders and providers and enlisting a culturally and linguistically specific committee composed of community leaders to consult during the research development process.  4. Knowledge of what problems are critical  5. Approaches that show that research results are mutually beneficial to both researcher and the study population identified by the study population  6. Empowerment is achieved through mutual benefit of the study | 1. Researchers could seek study population’s experiences of culture in relation to how they feel, think and behave within the phenomenon /area of interest. (contextuality)  2. Researchers conduct the literature review as a starting point to look for voices which then be verified by the study group. (contextuality)  3. Researcher should engage with community leaders to provide culturally sensitive details to support research problem formulation. (relevance)  4.Include a study group participant in research. (contextuality)  5.Researchers to ask the community what forms of enrichment beyond monetary compensation participants would prefer. (reciprocation)  6. Researchers to demonstrate appreciation of power dynamics between them and participants. (empowerment) | 1. Is there a representation of a study population group to capture their voice in a form of qualitative/quantitative or mixed methodology?  2. Is there a literature review checklist?  3. Have the community leaders participated in the research problem formulation?  4. Has the ethics been considered when including a study group participant? (contextuality)  5.Have the researchers engaged with the community to decide about reciprocation? (reciprocation)  6. Have the researchers been reflexive of power dynamics? (empowerment) |
|  | Context  Relevance | Im et al. (1999a) | 1. developing a historical context for the study participants, and systematically developing and maintaining a socio-cultural context for the research encounter during every phase of the research process;  2. Any physiological event occurring within socio-cultural context needs to consider socio-cultural context , and cultural meanings should be considered along with physiological events.  Check if the historic and socio-cultural context are included which contradicts positivistic assumptions and objective reality. Lack of consideration of context can sometimes mislead to misinterpretation of research findings.  1.The research questions which reflect a study group’s interests and issues can be developed by health care providers based on literature review  2. Without understanding of the context and cultural heritage  the findings can be simplified  3. When considering contextual factors, under-reporting and/ or ignorance of symptoms may be other interpretations of the findings.  4. Establishing relevance or the research questions reflecting a population's issues and interests in improving their lives. Can be developed by health care providers largely based on literature review and some quantitative work  5. Can measurement scales developed with Western populations can adequately measure symptoms of  Asian populations? A symptom which is significant in one culture, can be ignored or disregarded in a different culture due to religion, privacy of body etc. | Repetition of points 2 (contextuality), point 1(relevance)  7. Researchers to consider the use of interpretation and /or translation services. (Language) | Repetition of points 2 (contextuality), point 1(relevance)  7. Have the researchers used the interpretation/translation services? (Language) |
|  | Context  Language | Im et al. (1999b) | 1. Information on sociodemographic characteristics does not provide any detail on the participants until the meanings of these characteristics are uncovered by participants;  2. It is important to gain information on the meaning participants attribute the experiences provides;  3. Important to use qualitative research methods to explore the meanings participants attribute to the experiences  4. Virtually all of the reviewed studies included information  on sociodemographic characteristics of  their research participants; however, the number  of the studies that focused on the meaning of these  characteristics and on uncovering women’s individual  experiences and the meaning that they  attribute to their menopausal experience is still  minimal. More recently, some researchers (five  studies among the 158 studies) have used qualitative  research methods to explore women’s  menopausal experience within the social context  of their lives, and therefore provide a rich description  of menopausal experiences and factors influencing  them  5. Interpretation of symptoms and terms is critical instead of literal translation |  |  |
|  | Contextuality  Relevance  Reciprocation | Meleis (1996), | 1. Absence of participants’ knowledge leads to marginalisation of populations and to stereotyping of groups  2. The extent to which the problem area and the research questions are considered significant and meaningful for their health  3. All parties involved meet their own goals from the research process and through the research findings. The researcher’s goal is to gain understanding related to the area of investigation, to answer research questions, and to identify patterns and processes. | Repetition of points 1-7 | Repetition of points 1-7 |
|  | Contextuality  Relevance | Jacobson (2005) | 1. Important to create knowledge of participants’ lifestyles, their historical and sociopolitical circumstances affecting them  2. Significance of a topic for study group is guided by cultural knowledge rather than by importance of influence on their life and health | Repetition of point 1 (contextuality) | Repetition of point 1 (contextuality) |
|  | Contextuality | Meleis (1998) | 1.To view study group through their cultural premises may lead to researchers justifying their neglect arguing that there is way to change their patterns of behaviour.  2.Researchers need to reduce risk of marginalizing the populations by applying a diversity framework including age, sexual orientation, cultural heritage, social class, work situation and gender inequity. | Repetition of point 1 (contextuality)  8. Researchers to consider researcher and participant cultural matching. (relevance) | Repetition of point 1 (contextuality)  8. Have the researchers considered researcher and participant cultural matching? |
|  | Contextuality  Relevance  Social Justice and Equity Orientation  Conceptual Clarity  Mutual Trust and Respect | Meleis (1999) | 1. Context awareness is underpinned by embodied valuations of diversity at all levels and in all areas of lives by manifesting a sense of challenge by diversity and a lack of threat from differences.  2. Cultural competency does not mean constant interpretation of symbols but instead is challenged by teaching, researching or caring for people who are different from them  3. It is impossible to have knowledge about various cultures but it is possible to have knowledge of different and similar patterns of responses while considering uniqueness of individuals as a product of their own life experiences. | 9. The researchers need to consider gaining cultural competency training. (contextuality) | 9. Have the researchers gained cultural competency training? (contextuality) |
|  | Contextuality  Awareness of identity and power differentials | Mendias (2001) | 1. Knowing participants’ lifestyles and circumstances helps to avoid stereotyping and place the study in context  2. Every research phase must cultivate a sociocultural context  3. Being attuned to differences in identity and power can shed light to awareness of identity and power differentials | Repetition of points 1, 2 and 6 (relevance) | Repetition of points 1, 2 and 6 (relevance) |
| **2. Recruitment** | Communication style  Awareness of Identity and Power Differences  Disclosure Time | Casado et al. (2012) | 1.Cultural Responsiveness Beyond Language Matching: Instead of only hiring bilingual staff who speak the participants’ languages, the team ensures these staff also understand the community’s unique socioeconomic backgrounds and immigration histories.  2. Researchers may then need to act as “cultural  brokers” between the ethnic minority community and the research establishment and may have to educate the institutional review board members regarding culturally responsive protocol that also meets the ethical standards of research.  3. Avoid secrecy  4. Be flexible and think about ways to reimburse participants’ time | Repetition of point 9 (communication style)  10. Researchers to develop a communication strategy with a community in focus (communication style)  11. Researchers may need to consider acting as “cultural brokers” between ethnic minority community and the research establishment (Awareness of Identity and Power Differences  )  Repetition of point 1 (disclosure)  12. Researchers need to be flexible in considering relevant types of reimbursement for participants’ time. (Time) | Repetition of point 9 (communication style)  10. Has a communication strategy been described? (communication style)  11. Have the researchers described how they have acted as brokers? (Awareness of Identity and Power Differences  )  Repetition of point 1 (disclosure)  12. Have the researchers considered different types of reimbursement? (Time) |
|  | Contextuality  Language  Communication style | Im et al. (1999a) | 1. developing a historical context for the study participants, and systematically developing and maintaining a  socio-cultural context for the research encounter  during every phase of the research process  providing evidence of critical understanding of preferred communication styles for the research participants and their communities  2. Consider the differences in the degree of emotions in the language (in comparison with English) ( for example: when describing pain)  3. assumption that a researcher should be in a value-free, neutral, and objective position, subsequently emphasizing a distance between the researcher and the research participants | 13. Researchers to have people’s input in how we invite and offer participants to undertake data collection (contextuality)  14. Multicultural PPI group with a representative of certain culture to sense check the approach (language)  15. Researchers to consider the use of interpretation and /or translation services. (communication style) | 13. Have the researchers described how PPIE informed the recruitment strategy? (contextuality)  14. Have the researchers included a multicultural PPI group? (contextuality)  15. Have the researchers used the interpretation/translation services? (communication style) |
|  |  | Im et al (1999b) |  | 16. Researchers to have a more representative picture of the study population (relevance) | 16. Have the researchers used the research describing the approach to sampling? |
|  | Awareness of Identity and Power Differentials | Meleis (1996) | 1. less distance between the researcher and the participants  2. participants can exercise the power to dictate the research questions or to refuse to participate in the research project.  3. demonstrate that the researcher is cognizant of the power differential and that the levels of hierarchical power are acknowledged  4. working together to increase control over one life events  5. Evidence of time frame and the dimensions of time that allow the researchers to meet their own as well as the participants’ goals.  6. well-defined sense of the self as manifested in being able to question the research process and to experience a sense of freedom and options in modifying any parts of the process.  7. flexible approach to time  8. Awareness of the meaning of time and how it is used in research report | Repetition of point 6. |  |
|  | Communication styles  Awareness of identity and power differentials  Disclosure  Reciprocation  Empowerment  Time  Ethics | Jacobson (2005) | 1. Awareness of participants’ preferred language, use of symbols and oral, written and scaled responses  2. Developing horizontal relationships  3. Awareness of secrecy, trust and rapport  4. Research is recognised as an exchange with incentives provided  5. Participants receive some lasting sense of control over a problem or some skill for improving their health)  6. researchers collect data at participants’ convenience, discusses how different time perspectives may have influenced study process or results  7. Investigators need to consider how to respond to personal dilemmas in which the participants’ cultural beliefs and norms differ markedly from those of the investigator | 17. Researchers to consider strategies for empowering study participants that go beyond incentivisation (reciprocation)  18. Ethics should be discussed through the lens of ethnicity and culture. (ethics, disclosure) | 17. Researchers to describe and rationalise strategies of empowering participants (reciprocation)  18. Has the ethics been discussed through the lens of ethnicity and culture? (ethics) |
| **3. Measurement** | Contextuality  Communication style  Time | Casado et al. | 1.The translation is an intricate process that requires an understanding of the cultural meaning of the concept being studied i.e. developing linguistic equivalency  2.Application of the translation technique by Brislin (1970) which consists of a series of translations and back-translations of the original instrument by bilingual individuals.  3. It is essential that translation be conducted by at least two bilingual individuals who understand culturally specific language use and expressions in both English and the  4. If for bilingual interviewers receive extensive training, then the trained bilingual interviewers can conduct data collection entirely without the participation of the nonbilingual researcher target language. | 19. Researchers to consider the use of interpretation and /or translation services (contextuality)  20. Researchers may receive extensive training so that they can conduct data collection without the participation of nonbilingual researcher. (communication style)  21. Researchers need to consider resources e.g. time when analysing the data. (time) | 19. Have the researchers used the interpretation/translation services? (contextuality)  20. How does the researcher articulate that they have capability to translate data?(communication style)  21. Have the research team articulated the use of resources e.g. time? (time) |
|  | Contextuality | Im et al. (1999a) | 1. Developing a historical context for the study participants, and systematically developing and maintaining a  socio-cultural context for the research encounter  during every phase of the research process  2. providing evidence of critical understanding  of preferred communication styles for the research participants and their communities  3. uncovering participants' experiences in ways  that are authentic to the authors and understandable to the audience;  4. Without attention to the socio-cultural context there is a risk of cultural stereotyping of system reporting  5. Measurements used need to be based on heterogeneous samples | 22. Measurements used need to be designed and deployed with heterogeneous representatives. (contextuality)  23. Researchers may consider the nature of authentic experiences that are easily stigmatised and hidden under the veil of physical symptoms such as headache, loss of appetite, general malaise, muscle-ache or rheumatic pain. (contextuality) | 22. Have the measurements used been designed and deployed with heterogeneous representatives? (contextuality)  23. Have the researchers considered the possibility of authentic experiences hiding under the physical symptoms? (contextuality) |
|  | Contextuality  Communication style | Im et al. (1999b) | 1. New measurement methods have been developed based on simple literature reviews and used without adequate reliability  2. Establishing horizontal relationships: Should such a horizontal relationship be insured when using these scales? With the assumption of objectivity, can we ensure obtaining relevant and authentic data? If the answer is `no', how can we use a scale while minimizing power imbalance? If the answer is `yes,' then how can we overcome the paradox inherent in the utilization of a scale while ensuring horizontal relationships | 24. Researchers need to consider applicability of existing tools in target populations (contextuality) | 24. Have the researchers considered the use of tools in relation to the study population groups? (contextuality) |
|  | Contextuality  Communication style | Jacobson (2005) | Consider participants’ mobility and flexibility in leaving their homes (some women cannot leave without male escort)  Appropriate use of translation and interpretation, recognition of group’s difficulty with Likert-type scales | 25. Researchers could use PPI-informed statements for data collection. (contextuality)  26. Researchers need to consider applicability of existing tools in target populations. (communication style) | 25. Have researchers used PPI-informed statements for data collection? (contextuality)  26. Researchers need to consider applicability of existing tools in target populations? (communication style) |
|  | Contextuality | Meleis (1996) | 1. A qualitative research design might not be the only choice of framework for research projects in which the culture of the population is only one consideration, while the central focus of the study is driven by the mission of health professionals | Repetition of point 16 |  |
|  | Communication style | Meleis (1998) | 1.Marginalized populations tend to have their own approach and their own silent language that they protect and shield from the majority. The interpretation of the meanings of the experiences of the marginalized people may be limited in depth. | 27. Researchers could to seek study population’s experiences of culture in relation to how they feel, think and behave within the phenomenon /area of interest. (communication style) | 27. Is there a representation of a study population group to capture their voice in a form of qualitative/quantitative or mixed methodology? (communication style) |
| **4. Data Analysis & Interpretation** | Language | Casado et al. (2012) | 1.Insider researchers have the ability to potentially translate and analyze data on their own, which is advantageous, some warn of the threat of bias in this process | 28. Researchers to consider the use of interpretation and /or translation services. (language) | 28. Have the researchers used the interpretation/translation services? (language) |
|  | Contextuality | Im et al. (1999a) | 1.The validation of the questions included in measurement scales through focus groups, explorations, use of open ended questions, attention to language use, and knowledge of linguistic nuances need to be incorporated in pilot studies to enhance the development and use of culturally competent questions. | 29. Researchers could seek study populations’ experiences and expressions of culture in relation to thoughts, feelings and behaviour within the phenomenon of interest  30. Researchers could include a strategy of comparator groups to identify cultural similarities and differences  Repetition of points 1, 16 | 29. Is there a representation of a study population group to capture their voice in a form of qualitative/quantitative or mixed methodology?  30. Have researchers included a strategy of comparator groups to identify cultural similarities and differences? |
|  | Communication style | Im et al. (1999b) | 1.  The  passive relationships of participants may contribute to the overestimation  and/or the underestimation of  menopausal symptoms among Korean women. | 31. Researchers could seek study population’s experiences of culture in relation to thoughts, feelings and behaviour within the phenomenon of interest. (communication style) | 31. Has the nature of relationships between participants and a researcher been considered? (communication style) |
|  | Communication style | Jacobson (2005) | 1.Recognition of group’s difficulty with Likert-type scales | Repetition of point 1 |  |
|  | Language | Meleis (1996) | 1.A culturally competent person is one who is aware of how being different from the norm (language, education, clothing, sexual orientation) could be marginalizing and how marginalization may influence patterns of seeking and receiving care. | 32. Researchers need to include a reflective section on cultural competency. (language) | 32. Have the researchers included a reflective section on cultural competency? (language) |
|  | Contextuality Relevance  Language | Meleis (1998) | 1.Analyses and interpretations of the findings demonstrated that to develop some understanding about these populations, an awareness of history and context for marginalization should be developed. We had to truly come in touch with the women’s sufferings, with the ways by which others had stereotyped them, and with the processes used to differentiate them from mainstream. | 33. Researchers are advised to consider co-analysis of data together with a study population group. (contextuality; relevance)  34. Researchers are advised to ground their data analysis and interpretation within the specific population or cultures of interest. (contextuality; relevance)  Repetition of point 28 and 32 | 33. Have the researchers conducted co-analysis of data with a study population group? (contextuality; relevance)  34. Is there sufficient description of specificities (what is generalisable and what is not?)? (contextuality; relevance) |
|  | Language | Meles (1999) | 1.A culturally competent person is one who is aware of how being different from the norm (language, education, clothing, sexual orientation) could be marginalizing and how marginalization may influence patterns of seeking and receiving care. | 35. Researchers need to include a reflective section on cultural competency. (language) | 35. Have the researchers included a reflective section on cultural competency?(language) |
|  | Ethics  Time  Disclosure | Mendias (2001) | 1.Attuned to differences in identity and power; participants have opportunities to dictate study questions or refuse participation  2. Project viewed with flexible view of time, time adjusted to meet researchers’ or participants’ goals or to enhance reciprocity, disclosure, or empowerment  3. Disclosure includes sensitivity to secrecy and privacy issues and efforts to build trust so respondents feel able to respond freely or to decline response (Meleis, 1996). Measures for this criterion are (1) project approval and related study protocols, and (2) efforts to build community trust  4. Course philosophy Study protocols Adherence to Institutional Review Board requirements  5. Study protocols and interview practices  Relevance: The second means to determine that the research was viewed as relevant to the community was through participants’ comments about the study, which have been routinely requested and recorded at the conclusion of each interview | 36. Ethics should be discussed through the lens of ethnicity and culture. (ethics)  Repetition of points 18, 21 | 36. Has the ethics been discussed through the lens of ethnicity and culture? (ethics) |
| **5. Dissemination** | Reciprocation  Empowerment | Casado et al., 2012 | 1.Follow-through in sharing of study results and long-term commitment from researchers can often result in distrust among community members, especially those who are socially isolated from mainstream establishments, such as language minorities. | 37. Study findings be disseminated to stakeholders at all levels. (reciprocation)  38. Researchers could use various dissemination strategies, such as press releases, summary reports, research brief brochures, policy briefs, study newsletters, community agency publications and Web sites, etc. (reciprocation)  39. Researchers could consider diverse ways of sharing study results bearing in mind populations which are socially isolated from mainstream establishments. (empowerment) | 37. Have the findings been disseminated at all levels? (reciprocation)  38.Have the researchers demonstrated diverse ways of dissemination? (reciprocation)  39.Have the researchers had PPIE input?(empowerment) |
|  | Contextuality | Im et al., 1999a | 1. Developing a historical context for the study participants, and systematically developing and maintaining a socio-cultural context for the research encounter during every phase of the research process;  2. Demonstrating ¯flexibility in the research process, time lines, and researcher and participants encounters.  Example: Since menopausal status is not static,  but dynamic, a woman who was pre-menopausal can  be peri-menopausal after just one month (Kaufert et  al., 1988). Consequently, time delay in data collection  can evoke a bias | 40. Researchers could seek study population’s experiences of culture in relation to thoughts, feelings and behaviour within the phenomenon of interest in the dissemination strategy. (contextuality) | 40. Is there a representation of a study population group to capture their voice in a form of qualitative/quantitative or mixed methodology? (contextuality) |
|  | Empowerment  Time | Jacobson, 2005 | 1. Participants receive some lasting sense of control over a problem or some skill for improving their health  2. Research team is aware of group’s perspectives on time and accommodates them | 41. Researchers to disseminate findings in a meaningful way to participants so that study population group benefits from it (empowerment)  Repetition of point 21 | 41. Have the researchers been able to measure value from the dissemination strategy? (empowerment)  Repetition of point 21 |
|  | Disclosure  Relevance  Communication style | Meleis, 1996 | 1. Secrecy is a property of marginalization. Vulnerable groups may tend to keep their marginalized identities a secret and to attempt to pass as mainstream.  2. Demonstrate how the research questions, the interpretations, and the dissemination of data were informed and influenced by the community itself. | Repetition of points 1, 2, 3, 18 | Repetition of points 1, 2, 3, 18 |
|  | Reciprocation | Mendias, 2001 | 1. Philosophy and projects were congruent with the criterion of reciprocation. Our projects pursued a mutually positive purpose—to benefit the community while meeting the service goals of collaborating service institutions’ and our universities’ educational, research, and service goals | Repetition of point 17 | Repetition of point 17 |

1. Page MJ, McKenzie JE, Bossuyt PM, Boutron I, Hoffmann TC, Mulrow CD, et al. The PRISMA 2020 statement: an updated guideline for reporting systematic reviews. BMJ 2021;372:n71. doi: 10.1136/bmj.n71 [↑](#footnote-ref-1)
