# Supplementary material for: Cultural Competency in Research: A Practical Framework for Use by Researchers, Policymakers, Community Leads and Others When Working With People From Diverse Groups
Source: Health Expect. 2026 Jan 13;29(1):e70544. doi: 10.1111/hex.70544 (PMC12796843; doi:10.1111/hex.70544)
Supplement: Supplementary file 2 — Table 1: Coding details of workshop panellists. [file HEX-29-e70544-s002.docx]

**Supplementary File 3**

Table 1: Coding details of workshop panellists

| Workshop panellist | Years of experience | Coding |
| --- | --- | --- |
| Community lead 1 | 3-4 | CL1 |
| Community lead 2 | 3-4 | CL2 |
| Community lead 3 | 5-10 | CL3 |
| Community lead 4 | More than 10 years | CL4 |
| Researcher 1 | 3-4 | R1 |
| Researcher 2 | 3-4 | R2 |
| Researcher 3 | 5-10 | R3 |
| Researcher 4 | 5-10 | R4 |
| Researcher 5 | 5-10 | R5 |
| Researcher 6 | 5-10 | R6 |
| Policy maker 1 | More than 10 years | PM1 |
| Translator/Interpreter 1 | 4-5 | T/I1 |
| Translator/Interpreter 2 | 4-5 | T/I2 |
| Translator/Interpreter 3 | 4-5 | T/I3 |
