# Supplementary material for: Cultural Competency in Research: A Practical Framework for Use by Researchers, Policymakers, Community Leads and Others When Working With People From Diverse Groups
Source: Health Expect. 2026 Jan 13;29(1):e70544. doi: 10.1111/hex.70544 (PMC12796843; doi:10.1111/hex.70544)
Supplement: Supplementary file 3 — Table 1: Process of reaching consensus across modified Delph. [file HEX-29-e70544-s001.docx]

Supplementary file 2: Process of reaching consensus across modified Delphi

**Table 1: Process of reaching consensus across modified Delphi**

| **Research stage 1: Formulating the area of research focus and interest** | | | | | | | | | | |  |
| --- | --- | --- | --- | --- | --- | --- | --- | --- | --- | --- | --- |
| **Criteria** | **Recommendations** | **Round 1** | **Round 2** | **Workshop** | **Criteria** | **Measures** | **Round 1** | **Round 2** | | **Workshop** |  |
|  |  | **% of important rating and median of whole group** | **% of important rating and median whole group** |  |  |  | **% of important rating and median of whole group** | **% of important rating and median whole group** | | **Consensus reached** |  |
| Language | Researchers could enquire about the need for interpretation and /or translation services | Consensus reached by 88.5%, median 5 | Consensus reached in Round 1 | Panel members reached consensus on the recommendation after reframing it: Researchers should enquire about the need for using a variety of translation and interpretation services (such as transcription of interviews or parts of it in original language, using lay researchers’ services etc) as well as transcription for people with accessibility needs. | Language | Have researchers engaged with and used the qualified interpretation/translation services? | Consensus reached by 92%, median 5 | Consensus reached in Round 1 | | Panel members reached consensus on the recommendation after reframing it:  How did the researchers engage with and use lay researchers, the qualified interpretation/translation services and considered variety of ways of engaging with those through transcribing and interpreting data as well as transcribing it for people with accessibility needs? |  |
| Contextuality | Researchers should ensure their  research focus is sensitive to the  community of interest by considering  intersecting factors such as age,  generation, sexual orientation,  cultural heritage, social class, work  conditions, and gender inequality. | Consensus reached by 88.5%, median 5 | Consensus reached in Round 1 | Panel members reached consensus on the recommendation after reframing it: Researchers should ensure their  research focus is sensitive to the  community of interest by considering  intersecting factors such as age,  generation, sexual orientation,  cultural heritage, social class, work  conditions, and gender inequality. | Contextuality | Have the researchers aligned their  research focus with sensitivity to  intersectional factors such as age,  generation, sexual orientation, cultural  heritage, social class, work conditions,  and gender inequality within the  community of interest? | Consensus reached by 88%, median 5 | Consensus reached in Round 1 | | Panel members reached consensus on the measure after reframing it: Have the researchers aligned their  research focus with sensitivity to  intersectional factors such as age,  generation, sexual orientation, cultural  heritage, social class, work conditions,  and gender inequality within the  community of interest? |  |
| Contextuality | Researchers need to consider building direct relationships with the community in focus. | New statement added | Consensus reached in Round 2 by 91.7%, 5 | Consensus reached during the workshop after rewording: Researchers should consider creating capacity within communities of interest | Contextuality | Have researchers considered ways of building direct relationships with the community in focus by activities other than research-related? | No consensus reached | No consensus reached | | Consensus reached during the workshop, reworded:  Have the researchers explored ways to  build capacity within the community of  interest through activities beyond the  research itself? |  |
| Contextuality | Researchers could seek to study diverse populations’ experiences of culture about how they feel, think, express and behave within the phenomenon /area of interest and how this may underpin their perspectives, expressions and behaviours. | No consensus reached | Consensus reached in Round 2 by 87.5%,5 | Consensus reached after reframing the recommendation during the workshop: Researchers should explore how  diverse populations experience  culture—how they feel, think, express  themselves, behave, and carry out  daily practices—and how these  experiences shape their perspectives  and actions within the area of  interest. | Contextuality | Is there representation of voice and lived experience of that is inclusive of the entire study population? | *Consensus not reached*, median 5 | Consensus reached by 95%, median 5 | | Panel members reached consensus on the measure after reframing it: Has and in what way the researcher  learned about and reported on diverse  voices and lived experiences of the  community in focus? |  |
| Relevance | Researchers could include a patient/ public member in the research team to guide culturally sensitive problem formulation | *Consensus not reached,* median 4 | Consensus reached by 95.8%, median 4 | Consensus reached on rewording the statement to: Researchers should include a members of the community/ public member in the research team to guide culturally sensitive problem formulation | Relevance | Does the research team include a study group representative? | No consensus reached | No consensus reached | | Panel members reached consensus on the measure after reframing it: Does the research team include a members of the community /public member in the research team to guide culturally sensitive problem formulation? |  |
| Contextuality | Researchers could undergo cultural competency training | *Consensus not reached,* median 4 | *Consensus not reached*, median 5 | Consensus reached on rewording the statement to: Researchers should engage in  ongoing cultural humility training that  encourages self-reflection on their  attitudes, positionality, and openness  to cultural differences, alongside  cultural competency training focused  on the specific community of interest. | Contextuality | Have the researchers gained cultural competency training? | No consensus reached | No consensus reached | | Consensus reached on rewording the statement to: Have the researchers engaged in ongoing  cultural humility training—focusing on  their attitudes, positionality, and  openness to cultural differences—along  with cultural competency training specific  to the community of interest? |  |
| Empowerment | Researchers could show evidence to demonstrate their appreciation of power dynamics between them and different groups and communities involved. | No consensus reached | No consensus reached | Consensus reached during the workshop, reworded:  Researchers should show evidence to demonstrate their appreciation of power dynamics between them and different groups and communities involved. | Empowerment | Have the researchers provided an opportunity for participants to freely share their ideas in relation to formulating the area of research focus and interest? | *Consensus not reached*, median 5 | Consensus reached by 95%, median 5 | |  |  |
| Language | Researchers could consider the need for additional funding dedicated to cover costs for translation and interpretation | New statement added | Consensus reached by 91.7%, median 5 | Consensus reached on rewording the statement to: Researchers should consider costs and benefits of using a variety of translation and interpretation services (such as transcription of interviews or parts of it in original language, using lay researchers’ services etc) | Language | Have the researchers considered including the additional costs covering for translation and interpretation in the funding bid? | No consensus reached | No consensus reached | | Consensus reached during the workshop after rewording:  Have researchers considered costs and benefits of using a variety of translation and interpretation services (such as transcription of interviews or parts of it in original language, using lay researchers’ services etc) |  |
| Relevance | Researchers could engage with community leaders who can provide culturally sensitive information to support research problem formulation. | *Consensus not reached*, median 5 | *Consensus reached* by 87.5%, median 4 | Consensus reached on rewording the statement to: Researchers should equally engage with diverse members, leaders and experts in their field within the community of interest who can provide culturally sensitive information to support research problem formulation. | Relevance | How have community leaders participated in/contributed to the formulation of the area of research focus and interest? | No consensus reached | No consensus reached | | Consensus reached at the workshop on rewording the statement to: How have diverse members, leaders and experts in their field within the community of interest participated in/contributed to the formulation of the area of research focus and interest? |  |
| Reciprocation | Researchers could ask communities involved in the research what forms of enrichment beyond monetary compensation participants would prefer. | No consensus reached | No consensus reached | Consensus reached at the workshop after rewording the recommendation to: Researchers should work with  members of the diverse community  of interest to discuss and agree on  fair compensation strategies for  participants’ involvement in the  research. | Reciprocation | Have the researchers engaged with the study population to discuss and agree the strategy of compensation for participants for their involvement in the research? | *Consensus not reached*, median 5 | *Consensus not reached*, median 4 | | Consensus reached on rewording the statement to: Have the researchers engaged with the  diverse community of interest to discuss  and agree the strategy of compensation  for participants’ involvement in the  research? |  |
| Relevance | Researchers should have a strategy to capture and monitor impact and value of patient and public involvement. | New statement added | Consensus reached by 91.7, median 5 | Stayed the same | Relevance | Do researchers have a strategy to capture and monitor impact and value of patient and public involvement? | *added* | Consensus reached, 91.7 | | Stayed the same |  |
| Contextuality | Researchers could conduct a literature review initially to establish which voices of the study population have already been explored. | No consensus reached | No consensus reached | No consensus reached | Contextuality | Do researchers conduct a literature review initially to establish which voices of the study population have already been explored? | No consensus reached | No consensus reached | | No consensus reached |  |
| Relevance, Awareness of Identity and Power Differentials | New: Researchers could consider development of a patient/public involvement strategy to ensure their impact is useful, productive, appropriate, and balanced in relation to ratio (PPIE/researchers) | New statement added | No consensus reached | No consensus reached | Relevance, Awareness of Identity and Power Differentials | Do researchers consider development of a patient/public involvement strategy to ensure their impact is useful, productive, appropriate, and balanced in relation to ration (PPIE/researchers)? | No consensus reached | No consensus reached | | No consensus reached |  |
| **Research stage 2: Recruitment** | | | | | | | | | | | |
| **Criteria** | **Recommendations** | **Round 1** | **Round 2** | **Workshop** | **Criteria** | **Measures** | **Round 1** | **Round 2** | **Workshop** | | |
|  |  | **% of important rating and median of whole group** | **% of important rating and median whole group** |  |  |  | **% of important rating and median of whole group** | **% of important rating and median whole group** | **Consensus reached** | | |
| Communication Style | Researchers could develop a communication strategy tailored with community involvement. | Consensus reached by 88.5%, median 5 | Consensus reached in Round 1 | Reworded at the workshop: Researchers should develop a  tailored communication strategy with  the community of interest. | Communication Style | Has a tailored communication strategy been described, implemented, undertaken and assessed? | Consensus reached by 95%, median 4 | Consensus reached in Round 1 | Reworded at the workshop: Have the researchers developed a communication strategy tailored with community involvement? | | |
| Language | Researchers could consider the use of interpretation and /or translation services | Consensus reached by 88.5%, median 5 | Consensus reached in Round 1 | Consensus reached on rewording the statement to: Researchers should consider costs and benefits of using a variety of translation and interpretation services (such as transcription of interviews or parts of it in original language, using lay researchers’ services etc) | Language | Have the researchers used the interpretation and/or translation services? | Consensus reached by 95%, median 5 | Consensus reached in Round 1 | Consensus reached on rewording the statement to: Have the researchers considered costs and benefits of using a variety of translation and interpretation services (such as transcription of interviews or parts of it in original language, using lay researchers’ services etc)? | | |
| Contextuality | Researchers could engage with a trusted/respected person within their field/community who can provide culturally sensitive information to support study recruitment. | *Consensus not reached*, median 5 | Consensus reached by 100%, median 4 | Consensus reached on rewording the statement to: Researchers should engage with a group of trusted/respected people within their field/community who can provide culturally sensitive information to support study recruitment. | Context | Have the researchers described how PPIE informed the recruitment strategy? | Consensus not reached | Consensus not reached | Consensus reached after rewording the statement to: Have researchers engaged with a group of trusted/respected people within their field/community who can provide culturally sensitive information to support study recruitment? | | |
| Relevance | Researchers could demonstrate how they have tailored recruitment and facilitated a diverse representation of the community | Consensus not reached | Consensus not reached | Consensus reached at the workshop, reworded: Researchers should demonstrate how they have tailored recruitment and facilitated a diverse representation of the community | Relevance | Have the researchers demonstrated how their recruitment strategy has facilitated a diverse insight of the study population? | Consensus not reached, Median 4 | Consensus reached by 95%, median 4 |  | | |
| Disclosure | Ethics should be discussed through the lens of ethnicity and culture including provision of culturally appropriate study explanations, assessing the cultural impediments to what researchers consider truly informed consent or the degree to which it is achieved, considering the ratio of risks to benefits in the research from the cultural perspective of the potential participants. | *Consensus not reached*, median 5 | Consensus reached by 95.8%, median 4 | Consensus reached on rewording the statement to: Ethics should be addressed through  the lens of ethnicity and culture by: (i)  ensuring ethics committees receive  cultural training, (ii) providing  culturally appropriate explanations of  the study, (iii) assessing cultural  barriers to achieving truly informed  consent, and (iv) evaluating the risk–  benefit ratio from the cultural  perspective of potential participants. | Disclosure | Has the ethics been discussed through the lens of ethnicity and culture? | Consensus not reached, median 4 | Consensus reached by 91%, median 4 | Consensus reached on rewording the statement to: Has ethics been considered and applied  through the lens of ethnicity and culture,  including: (i) cultural training for the ethics  committee, (ii) provision of culturally  appropriate study explanations, (iii)  assessment of cultural barriers to  achieving truly informed consent, and (iv)  evaluation of the risk–benefit ratio from  the cultural perspective of potential  participants? | | |
| Time | Researchers could spend time building trust with communities in focus and participants to promote engagement with the study. | New statement | Consensus reached by 95.8%, median 5 | Stayed the same | Time | Have researchers developed a strategy of building trust with communities in focus and participants to promote engagement with the study? | New statement | Consensus reached by 91%, median 5 | Stayed the same | | |
| Empowerment | Researchers could consider strategies for empowering participants that go beyond financial incentivisation (eg. Researchers to provide an ability to demonstrate connectedness to the research and a sense of freedom in modifying any parts of the process). | Consensus not reached | Consensus not reached | Consensus not reached | Empowerment | Have the researchers considered the strategies for empowering participants that go beyond financial incentivisation? | Consensus not reached | Consensus not reached | Consensus not reached | | |
| Time | Researchers could demonstrate flexibility in their strategy to reimburse and/or incentivise participants for their time | Consensus not reached | Consensus not reached | Consensus not reached | Time | Have the researchers demonstrated flexibility in their strategy to reimburse and /or incentivise participants for their time? | Consensus not reached | Consensus not reached | Consensus not reached | | |
| **Research stage 3: Measurement** | | | | | | | | | | | |
| **Criteria** | **Recommendations** | **Round 1** | **Round 2** | **Workshop** | **Criteria** | **Measures** | **Round 1** | **Round 2** | **Workshop** | | |
|  |  | **% of important rating and median of whole group** | **% of important rating and median whole group** |  |  |  | **% of important rating and median of whole group** | **% of important rating and median whole group** | **Consensus reached** | | |
| Language | Researchers to consider the use of interpretation and /or translation services | Consensus reached by 95.8%, median 4.5 | Consensus reached in Round 1 | Consensus reached on rewording the statement to: Researchers should consider costs and benefits of using a variety of translation and interpretation services (such as transcription of interviews or parts of it in original language, using lay researchers’ services etc) | Language | Have the researchers used the interpretation/translation services? | Consensus reached by 95%, median 5 | Consensus reached in Round 1 | Consensus reached on rewording the statement to: Have the researchers considered costs and benefits of using a variety of translation and interpretation services (such as transcription of interviews or parts of it in original language, using lay researchers’ services etc)? | | |
| Contextuality and relevance | Researchers could consider participants experiences of culture in relation to how they feel, think, express and behave and how this may influence data being collected in collaboration with appropriate stakeholders. | New statement | Consensus reached by 91.6%, median 4 | Consensus reached after rewording it: Researchers should consider participants experiences of culture in relation to how they feel, think, express and behave and how this may influence data being collected in collaboration with appropriate stakeholders. | Context and relevance | Have the researchers considered study populations’ experiences of culture in relation to how they feel, think, express and behave and how this may influence data being measured? | New statement | Consensus reached by 91%, median 4 | Consensus reached after rewording: Has the research learned about diverse voices and lived experiences of the community of interest? | | |
| Language | Researchers need to consider how communication is performed with non -native English speakers in order to ensure their involvement is facilitated in the research process. | New statement | Consensus reached by 94.8%, median 5 | Consensus reached on rewording:  Researchers should consider how they engage with non -native speakers in order to ensure their involvement is facilitated in the research process. | Language | Have the researchers considered how communication could be performed with non-native English speakers in order to engage them throughout the research process? | New statement | Consensus reached by 100%, median 5 | Consensus reached on rewording:  Have the researchers considered how they engage with non-native speakers in order to engage them throughout the research process? | | |
| Language | Researchers could conduct data collection within the language of the participant | Consensus not reached | Consensus not reached | Consensus not reached | Language | Have the researchers conducted data collection within the language of the participant? | Consensus not reached | Consensus not reached | Consensus not reached | | |
| Flexibility | Researchers could evidence that they have considered the resources, e.g. time, required to collect the data while respecting cultural preferences/ behaviours and accommodations | Consensus not reached | Consensus not reached | Consensus not reached | Flexibility | Have the researchers evidenced that they have considered the resources e.g. time, required to collect the data while respecting cultural preferences/behaviours and accommodations? | Consensus not reached | Consensus not reached | Consensus not reached | | |
| Relevance | Researchers need to consider applicability/validity of existing tools with the communities they work with. | Consensus not reached | Consensus not reached | Consensus not reached | Relevance | Have the researchers considered the applicability /validity of existing tools with the communities they work with? | Consensus not reached | Consensus not reached | Consensus not reached | | |
| **Research stage 4: Data analysis and interpretation** | | | | | | | | | | | |
| **Criteria** | **Recommendations** | **Round 1** | **Round 2** | **Workshop** | **Criteria** | **Measures** | **Round 1** | **Round 2** | **Workshop** | | |
|  |  | **% of important rating and median of whole group** | **% of important rating and median whole group** |  |  |  | **% of important rating and median of whole group** | **% of important rating and median whole group** | **Consensus reached** | | |
| Contextuality | Researchers could seek participants experiences and expressions of culture in relation to thoughts, feelings and behaviours and how this influences data interpretation | Consensus reached by 100%, median 5 | Consensus reached in Round 1 | Consensus reached after rewording: Researchers should explore and  report on how diverse populations  experience culture—how they feel,  think, express themselves, behave,  and carry out daily practices—and  how these experiences shape their  perspectives and actions within the  area of interest. | Context | Have the researchers considered study populations’ experiences of culture in relation to how they feel, think, express and behave and how this may influence data interpretation? | *Consensus not reached*, median 4 | Consensus reached by 100%, median 4 | Consensus reached after rewording: Has and in what way the researcher  learned about and reported on diverse  voices and lived experiences of the  community in focus? | | |
| Language | Researchers to consider the use of interpretation and /or translation services | Consensus not reached, median 5 | Consensus not reached, median 4 | Panel members reached consensus to remove the recommendation arguing that the co-analysis recommendation covers the translation and interpretation | Language | Have the researchers used the interpretation/translation services? | Consensus not reached, median 5 | Consensus not reached, median 5 | Panel members reached consensus to remove the recommendation arguing that the co-analysis recommendation covers the translation and interpretation | | |
| Contextuality | Researchers could consider co-analysis of data together with the community involved. | Consensus not reached, median 5 | Consensus not reached, median 4 | Consensus reached on rewording it:  Researchers should consider co-analysis of data together with the community involved and reimburse them for their time and input. | Contextuality | Have researchers considered co-analysis of data together with the community involved? | Consensus not reached | Consensus not reached | Panel members reached consensus on the measure to be included in the final list, matching statement, reworded:  Have researchers considered co-analysis of data together with the community involved followed by reimbursement for their time and input? | | |
| Relevance | Researchers could consider sense-checking the findings with communities in focus to ensure the analysis is calibrated to reflect the experiences expressed | New statement | Consensus reached by 91.6%, median 5 | Panel members voted to remove the recommendation due to difficulty of getting “consensus on sense checking from a diverse group of communities taking part in one study”. | Relevance | Have the researchers considered sense-checking the findings with communities in focus? | New statement | Consensus reached by 95%, median 5 | Panel members voted to remove the recommendation due to difficulty of getting “consensus on sense checking from a diverse group of communities taking part in one study”. | | |
| Relevance | Researchers could include a strategy of comparator groups to identify cultural similarities and differences | Consensus not reached | Consensus not reached | Consensus not reached | Relevance | Have the researchers included strategy of comparator group to identify cultural similarities and differences? | Consensus not reached | Consensus not reached | Consensus not reached | | |
| Empowerment | Researchers could consider including a reflexivity statement on cultural competency | Consensus not reached | Consensus not reached | Consensus not reached |  | Have the researchers considered including a reflexivity statement on cultural competency? | Consensus not reached | Consensus not reached | Consensus not reached | | |
| Relevance | Researchers could consider including a reflexivity statement on how their own culture and experiences influences the research process they are involved in | New statement | Consensus not reached | Consensus not reached |  | Have the researchers considered including a reflexivity statement? | New statement | Consensus not reached | Consensus not reached | | |
| Relevance | Researchers could consider applicability of research findings in relation to diverse community groups | New statement | Consensus not reached | Consensus not reached |  | Have the researchers considered applicability of research findings in relation to diverse community groups? | New statement | Consensus not reached | Consensus not reached | | |
| **Research stage 5: Dissemination** | | | | | | | | | | | |
| **Criteria** | **Recommendations** | **Round 1** | **Round 2** | **Workshop** | **Criteria** | **Measures** | **Round 1** | **Round 2** | **Workshop** | | |
|  |  | **% of important rating and median of whole group** | **% of important rating and median whole group** |  |  |  | **% of important rating and median of whole group** | **% of important rating and median whole group** | **Consensus reached** | | |
| Reciprocation | Researchers to disseminate findings in a meaningful way to participants so that the study population benefits from it | Consensus reached by 88.4%, median 5 | Consensus reached in Round 1 | Consensus reached after rewording: Study findings should be shared with  all levels of involvement—from  individual participants to the broader  community of interest—as well as  with diverse audiences. |  | Have the researchers been able to articulate and/or measure the value from the dissemination strategy? | Consensus not reached | Consensus not reached | Panel reached consensus, matching statement, reworded:  Have the researchers developed and included the dissemination plan agreed with the community of interest, participants, and patient and public involvement members? | | |
| Relevance | Researchers could consider alternative approaches that acknowledge diversity and culture of sharing study results that have been informed by participants experiences of thoughts, feelings and behaviours in relation to the studied phenomenon. | *Consensus not reached,* median 4 | Consensus reached by 94.8%, median 4 | Consensus reached after rewording: Researchers should explore and  report on how diverse populations  experience culture—how they feel,  think, express themselves, behave,  and carry out daily practices—and  how these experiences shape their  perspectives and actions within the  area of interest. |  | Have the researchers considered study populations’ experiences of culture in relation to how they feel, think, express and behave and how this may influence dissemination | Consensus not reached | Consensus not reached | Consensus reached at the workshop as a matching statement, reworded: Has and in what way the researcher  learned about and reported on diverse  voices and lived experiences of the  community in focus? | | |
| Empowerment and relevance | Study findings could be disseminated to levels of involvement (participant to communities) at a range of audiences. | Consensus not reached | Consensus not reached | Consensus reached at the workshop as a matching statement, reworded: Study findings should be disseminated to levels of involvement (participant to communities) at a range of audiences. | Empowerment and relevance | Have the researchers developed and included the dissemination plan agreed with participants, PPIE members as part of their involvement? | New statement | Consensus reached by 95%, median 4 |  | | |
