# Supplementary material for: Cultural Competency in Research: A Practical Framework for Use by Researchers, Policymakers, Community Leads and Others When Working With People From Diverse Groups
Source: Health Expect. 2026 Jan 13;29(1):e70544. doi: 10.1111/hex.70544 (PMC12796843; doi:10.1111/hex.70544)
Supplement: Supplementary file 4 — Supporting File 4: The final framework. [file HEX-29-e70544-s003.docx]

Supplementary File 4: The final framework

| Research Stage 1: Formulating the Area of Research Focus and Interest | | | |
| --- | --- | --- | --- |
| N | Criteria | Recommendation | Measure |
| 1 | Contextuality | Researchers should ensure their  research focus is sensitive to the  community of interest by considering  intersecting factors such as age,  generation, sexual orientation,  cultural heritage, social class, work  conditions, and gender inequality. | Have the researchers aligned their  research focus with sensitivity to  intersectional factors such as age,  generation, sexual orientation, cultural  heritage, social class, work conditions,  and gender inequality within the  community of interest? |
| 2 | Contextuality | Researchers should consider creating capacity within communities of interest. | Have the researchers explored ways to  build capacity within the community of  interest through activities beyond the  research itself? |
| 3 | Contextuality | Researchers should explore how  diverse populations experience  culture—how they feel, think, express  themselves, behave, and practice  daily life—and how these experiences  shape their perspectives and actions  within the area of interest. | Has and in what way has the researcher  learned about diverse voices and lived  experiences of the community of interest? |
| 4 | Contextuality | Researchers should engage in  ongoing cultural humility training that  encourages self-reflection on their  attitudes, positionality, and openness  to cultural differences, alongside  cultural competency training focused  on the specific community of interest. | Have the researchers engaged in ongoing  cultural humility training—focusing on  their attitudes, positionality, and  openness to cultural differences—along  with cultural competency training specific  to the community of interest? |
| 5 | Relevance | Researchers should have a strategy to capture and monitor impact and value of patient and public involvement. | Have researchers developed a strategy to capture and monitor impact and value of patient and public involvement? |
| 6 | Relevance | Researchers should equally engage with diverse members, leaders and experts in their field within the community in focus who can provide culturally sensitive information to support research problem formulation. | How have diverse members, leaders and experts in their field within the community in focus participated in/contributed to the formulation of the area of research focus and interest? |
| 7 | Relevance | Researchers should include members of the community/ public member in the research team to guide culturally sensitive problem formulation. | Does the research team include members of the community /public member in the research team to guide culturally sensitive problem formulation? |
| 8 | Language | Researchers should consider costs and benefits of using a variety of translation and interpretation services (such as transcription of interviews or parts of it in original language, using lay researchers’ services etc). | Have researchers considered costs and benefits of using a variety of translation and interpretation services (such as transcription of interviews or parts of it in original language, using lay researchers’ services etc)? |
| 9 | Language | Researchers should enquire about the need for using a variety of translation and interpretation services (such as transcription of interviews or parts of it in original language, using lay researchers’ services etc) as well as transcription for people with accessibility needs. | How did the researchers engaged with and used all of that interpretation with lay researchers, the qualified interpretation/translation services and considered variety of ways of engaging with those through transcribing and interpreting data as well as transcribing it for people with accessibility needs? |
| 10 | Empowerment | Researchers should show evidence to demonstrate their appreciation of power dynamics between them and different groups and communities involved. | Have the researchers provided an opportunity for participants to freely share their ideas in relation to formulating the area of research focus and interest? |
| 11 | Reciprocation | Researchers should work with  members of the diverse community  of interest to discuss and agree on  fair compensation strategies for  participants’ involvement in the  research. | Have the researchers engaged with the  diverse community of interest to discuss  and agree the strategy of compensation  for participants’ involvement in the  research? |
| Research Stage 2: Recruitment | | | |
| 12 | Time | Researchers should spend time building trust with communities of interest and participants to promote engagement with the study. | Have researchers developed a strategy of building trust with communities of interest and participants to promote engagement with the study? |
| 13 | Contextuality | Researchers should engage with a group of trusted/respected people within thecommunity of interest who can provide culturally sensitive information to support study recruitment. | Have researchers engaged with a group of trusted/respected people within the community of interest who can provide culturally sensitive information to support study recruitment? |
| 14 | Relevance | Researchers should demonstrate how they have tailored recruitment and facilitated a diverse representation of the community of interest. | Have the researchers demonstrated how their recruitment strategy has facilitated a diverse insight of the community of interest? |
| 15 | Disclosure | Ethics should be addressed through  the lens of ethnicity and culture by: (i)  ensuring ethics committees receive  cultural training, (ii) providing  culturally appropriate explanations of  the study, (iii) assessing cultural  barriers to achieving truly informed  consent, and (iv) evaluating the risk–  benefit ratio from the cultural  perspective of potential participants. | Has ethics been considered and applied  through the lens of ethnicity and culture,  including: (i) cultural training for the ethics  committee, (ii) provision of culturally  appropriate study explanations, (iii)  assessment of cultural barriers to  achieving truly informed consent, and (iv)  evaluation of the risk–benefit ratio from  the cultural perspective of potential  participants? |
| 16 | Language | Researchers should consider costs and benefits of using a variety of translation and interpretation services (such as transcription of interviews or parts of it in original language, using lay researchers’ services etc) | Have the researchers considered costs and benefits of using a variety of translation and interpretation services (such as transcription of interviews or parts of it in original language, using lay researchers’ services etc)? |
| 17 | Communication Style | Researchers should develop a  tailored communication strategy with  the community of interest. | Has a tailored communication strategy been described, implemented, undertaken and assessed? |
| Research Stage 3: Measurement | | | |
| 18 | Contextuality and relevance | Researchers should consider participants experiences of culture in relation to how they feel, think, express and behave and how this may influence data being collected in collaboration with appropriate stakeholders. | Has and in what way the research learned about diverse voices and lived experiences of the community of interest? |
| 19 | Language | Researchers should consider costs and benefits of using a variety of translation and interpretation services (such as transcription of interviews or parts of it in original language, using lay researchers’ services etc) | Have the researchers considered costs and benefits of using a variety of translation and interpretation services (such as transcription of interviews or parts of it in original language, using lay researchers’ services etc)? |
| 20 | Communication style | Researchers should consider how they engage with non -native speakers to ensure their involvement is facilitated in the research process. | Have the researchers considered how they engage with non-native speakers to engage them throughout the research process? |
| Research Stage 4: Analysis and Interpretation | | | |
| 21 | Contextuality | Researchers should explore how  diverse populations experience  culture—how they feel, think, express  themselves, behave, and carry out  daily practices—and how these  experiences shape their perspectives  and actions within the area of  interest. | Has and in what way the researcher learned about diverse voices and lived experiences of the community of interest? |
| 22 | Relevance | Researchers should consider co-analysis of data together with the community of interest and reimburse them for their time and input. | Have researchers considered co-analysis of data together with the community of interest followed by reimbursement for their time and input? |
| Research Stage 5: Dissemination | | | |
| 23 | Reciprocation | Researchers should disseminate findings in an understandable, meaningful way to participants so that the study population benefits from it and the research results work for the community of interest. | Have the researchers considered meaningful and understandable ways of disseminating the research results so that it works for people and in a way that works for them? |
| 24 | Empowerment, Awareness of Identity and Power Differentials | Researchers should explore and  report on how diverse populations  experience culture—how they feel,  think, express themselves, behave,  and carry out daily practices—and  how these experiences shape their  perspectives and actions within the  area of interest. | Has and in what way the researcher  learned about and reported on diverse  voices and lived experiences of the  community in focus? |
| 25 | Empowerment, Awareness of Identity and Power Differentials | Study findings should be shared with  all levels of involvement—from  individual participants to the broader  community of interest—as well as  with diverse audiences. | Have the researchers developed and included the dissemination plan agreed with the community of interest,participants, and patient and public involvement members? |
